# TLR3 deficiency exacerbates the loss of epithelial barrier function during genital tract *Chlamydia muridarum* infection

**DOI:** 10.1371/journal.pone.0207422

**Published:** 2019-01-09

**Authors:** Ramesh Kumar, Haoli Gong, Luyao Liu, Nicole Ramos-Solis, Cheikh I. Seye, Wilbert A. Derbigny

**Affiliations:** 1 Department of Microbiology and Immunology, Indiana University School of Medicine, Indianapolis, Indiana, United States of America; 2 Xiangya Second Hospital, Central South University, Changsha, Hunan Province, People’s Republic of China; 3 Department of Cellular and Integrative Physiology, Indiana University School of Medicine, Indianapolis, Indiana, United States of America; University of Texas Health Science Center at San Antonio, UNITED STATES

## Abstract

**Problem:**

*Chlamydia trachomatis* infections are often associated with acute syndromes including cervicitis, urethritis, and endometritis, which can lead to chronic sequelae such as pelvic inflammatory disease (PID), chronic pelvic pain, ectopic pregnancy, and tubal infertility. As epithelial cells are the primary cell type productively infected during genital tract *Chlamydia* infections, we investigated whether *Chlamydia* has any impact on the integrity of the host epithelial barrier as a possible mechanism to facilitate the dissemination of infection, and examined whether TLR3 function modulates its impact.

**Method of study:**

We used wild-type and TLR3-deficient murine oviduct epithelial (OE) cells to ascertain whether *C*. *muridarum* infection had any effect on the epithelial barrier integrity of these cells as measured by transepithelial resistance (TER) and cell permeability assays. We next assessed whether infection impacted the transcription and protein function of the cellular tight-junction (TJ) genes for claudins1-4, ZO-1, JAM1 and occludin via quantitative real-time PCR (qPCR) and western blot.

**Results:**

qPCR, immunoblotting, transwell permeability assays, and TER studies show that *Chlamydia* compromises cellular TJ function throughout infection in murine OE cells and that TLR3 deficiency significantly exacerbates this effect.

**Conclusion:**

Our data show that TLR3 plays a role in modulating epithelial barrier function during *Chlamydia* infection of epithelial cells lining the genital tract. These findings propose a role for TLR3 signaling in maintaining the integrity of epithelial barrier function during genital tract *Chlamydia* infection, a function that we hypothesize is important in helping limit the chlamydial spread and subsequent genital tract pathology.

## Introduction

*Chlamydia trachomatis* is a gram-negative intracellular bacterium and the cause of the disease chlamydia, which is the most common sexually transmitted infection in the United States, with over 1.7 million cases reported in the US in 2017 alone [1]. Genital tract infections with *C*. *trachomatis* are associated with many acute syndromes including cervicitis, urethritis, and endometritis [2]. Complications from chronic infections include pelvic inflammatory disease (PID) and its sequelae of chronic pelvic pain, ectopic pregnancy, and tubal infertility [3]. Although *Chlamydia* is treatable with antibiotics, infected individuals are often asymptomatic; which facilitates the spread of the bacterium through further sexual contact. As a result, *Chlamydia* infections have continued to rise despite the implementation of screening and early intervention strategies [4]. The ultimate goal in developing more effective therapeutic measures against *Chlamydia* infection is to identify aspects of host immunity that will augment clearance of the pathogen while minimizing immune responses that lead to genital tract pathology.

As an obligate intracellular pathogen, Chlamydiae are known to interact with host-cell pattern recognition receptors (PRRs), including a variety of intracellular cytosolic receptors and Toll-like receptors (TLRs) [5–10]. TLRs are PRRs that recognize conserved microbial molecules or pathogen-associated molecular patterns (PAMPs) [11]. Stimulation of TLRs by chlamydial PAMPs triggers cytokine responses critical to the establishment of innate and adaptive immune responses [5, 7, 12–15]. It is critically important to identify the TLRs that induce the specific inflammatory mediators that cause scarring and fibrosis, and to define therapeutic approaches to prevent this process.

TLR3 is a receptor for double-stranded RNA (dsRNA) and is known to activate transcription of IFN-β via the adaptor protein Toll-IL-1 receptor (TIR) domain-containing adaptor molecule-1 (TICAM-1) [also called TIR-domain-containing adapter-inducing IFN-β (TRIF)] [16, 17]. TLR3 is expressed intracellularly and on the cell surface on human fibroblasts [17]; however, TLR3 has an exclusive intracellular expression in most other cell types [18–20]. TLR3 has been identified as the major MyD88-independent PRR stimulated in the type-1 IFN responses to many different viral infections due to its intracellular localization [21–26]. Conversely, its role in bacterial infection is poorly understood, particularly since bacteria are not known to possess a dsRNA moiety. We previously showed that *C*. *muridarum* infected murine oviduct epithelial (OE) cells secrete IFN-β in a mostly TLR3 dependent manner and that they demonstrate dramatic reductions in the syntheses of other inflammatory immune mediators in addition to IFN-β [6, 8]. Results from our recent *in vivo* study show that TLR3-deficient mice have significantly different levels of several key innate-immune factors secreted into their genital tracts during *C*. *muridarum* infection, and demonstrate altered recruitment of CD4^+^ T-cells to the reproductive tract when compared to wild-type control mice [27]. Because of this altered immune response to *C*. *muridarum* infection, we routinely observe higher bacterial burdens early and mid-infection, and more substantial reproductive tract pathology in the TLR3-deficient mice. These findings have proved our hypothesis that a less than optimal immune response in the TLR3-deficient mice increases the severity of *Chlamydia* infection, and provides an impetus to investigate putative mechanisms that are invoked to elicit the more progressive disease in the TLR3-deficient mice.

In this study, we further delineate the role of TLR3 in genital tract pathology associated with *Chlamydia* infection, by examining the impact that TLR3 signaling has on bolstering the host’s protective epithelial barrier that is designed to limit the spread of infection. Here, we show that *Chlamydia* has an impact on the stability and function of cellular TJs throughout infection in murine OE cells, and that TLR3 function significantly modulates this effect.

## Materials and methods

### Ethics statement

C57BL/6J (control) and TLR3-deficient mice were purchased from The Jackson Laboratory (Bar Harbor, ME) at 6–8 weeks of age. All mice were provided food and water *ad libitum* and kept on a standard 12-hour light/dark cycle. All mice were given at least a 1-week period where they were allowed to acclimate to their new environment. After acclimation, female C57BL/6NJ and TLR3-deficient mice were injected with 2.5 mg Depo-Provera after being briefly anesthetized with isoflurane before any experiment, and all mice were allowed to recover for no less than 1-week after the Depo-Provera treatment. To alleviate any possible distress, the mice were also briefly anesthetized with isoflurane prior to either intravaginal infection with *C*. *muridarum*, the insertion of calcium alginate swabs, or the insertion of aseptic vaginal sponges. All mice were monitored daily for lethargy, signs of vaginal bleeding, and death. None of the mice exhibited morbidity during these studies. All mice were euthanized by either exposure to isoflurane or inhalation of carbon dioxide, followed by exsanguination. The Indiana University Institutional Animal Care and Use Committee (IACUC) approved all experimental animal protocols. All care was used to ensure that steps were taken to ameliorate animal suffering in all work involved in the removal of genital-tract tissue.

### Cells and bacteria

The cloned oviduct epithelial cell lines OE-TLR3(-) and OE-129WT [6] were grown at 37°C in a 5% CO2 humidified incubator and maintained in epithelial-cell media: 1:1 Dulbecco’s modified Eagle medium:F12K (Sigma-Aldrich, St. Louis, MO), supplemented with 10% characterized fetal bovine serum (FBS) (HyClone), 2 mM L-alanyl-L-glutamine (Glutamax I; Gibco/ Invitrogen, Carlsbad, CA), 5μg of bovine insulin/ ml, and 12.5ηg/ ml of recombinant human FGF-7 (keratinocyte growth factor; Sigma) as previously described [6, 28].

*Mycoplasma*-free *C*. *muridarum* Nigg, previously known as *C*. *trachomatis* strain (MoPn), was grown and titrated in McCoy cells (ATCC) as described [28, 29]. Chlamydial elementary bodies (EBs) were harvested from infected cells, resuspended in SPG buffer (250mM sucrose, 10mM sodium phosphate, and 5mM L-glutamic acid, pH 7.2), and quantified on McCoy cells using the methodology described previously [8, 28, 30].

### Mice and infections

Wild-type control mice C57BL/6NJ [Stock No 005304] and TLR3-deficient mice B6N.129S1-Tlr3tm1Flv/J [Stock No 009675] were purchased from The Jackson Laboratory (Bar Harbor, ME) at 6–8 weeks of age. All mice were housed in Indiana University Purdue University-Indianapolis specific pathogen-free (SPF) facilities. Age-matched mice were used at 14–16 weeks for the experiments in this study. The Indiana University Institutional Animal Care and Utilization Committee (IACUC) approved all experimental protocols.

Infections of mice were done as described in [5] with some minor modifications. Groups of 6 mice (1 mock treated; 5 infected) were treated with 2.5mg of Depo-Provera (medroxyprogesterone acetate; Pfizer; New York, NY) in 0.1 ml saline one week before vaginal infection with 10^5^ IFU *C*. *muridarum* (approximately 100 times the ID50) in 10μl sucrose-phosphate-glutamic acid (SPG) buffer (250mM sucrose, 10mM sodium phosphate, and 5mM L-glutamic acid, pH 7.2). To procure genital tract tissue from the infected mice, we used the methodology described in [31] with some modification. Briefly, the entire genital tract was sterilely harvested from each mouse on either day 3, day 5, or day 7 post-infection. The segment encompassing the oviduct and uterine horn was carefully excised from each side of the genital tract, and both sides from the same mouse were combined to comprise the entire upper genital tract (UGT) tissue sample from that particular mouse. Tissue samples were quickly frozen at -80°C until the time of use.

*In vitro* infection of the oviduct epithelial cell lines OE129WT and OE129TLR3(-/-) C19 were performed as described in [6, 32]. Briefly, the OE cells were plated in their respective cell-culture plates and were infected when confluent. The cells were infected with an inoculum containing anywhere from 1 to 10 inclusion-forming units (IFU) of *C*. *muridarum*/ cell (depending upon the experiment), and the cell-culture plates were centrifuged 1000 x *g* for 1hr at room temperature in a table-top centrifuge to synchronize infection prior to being returned to the 37°C CO_2_ incubator.

### Measuring *C*. *muridarum* organism recovery from upper genital tract tissue

We used a similar method to monitor the upper genital tract infection in mice as described elsewhere [31]. To recover viable *Chlamydia* from the UGT of *C*. *muridarum* infected wild-type and TLR3-deficient mice, each mouse’s UGT tissue sample was homogenized in 300μl of SPG using a 2-ml Dounce tissue grinder (Sigma) and subsequently passaged through a 20-gauge syringe. After a brief sonication, the released chlamydial EBs were titrated on McCoy cell monolayers as described previously in [8, 28, 30]. Briefly, chlamydial elementary bodies harvested from infected tissue were serial diluted 1:10 in SPG and quantified as inclusion forming units (IFUs) per milliliter on McCoy cell monolayers. The total number of IFUs per ml was calculated based on the enumeration of fluorescent inclusion bodies in the cells whereby antibody specific for chlamydial LPS was used to detect chlamydial inclusions in the infected McCoy cells. Detection of the chlamydial LPS was done via Alexa Fluor 488 anti-mouse IgG secondary antibody (Invitrogen/Life Technologies; Carlsbad, CA), and immuno-staining results were scanned and recorded by EVOS imaging system (Thermo-Fisher, Pittsburgh, PA).

### Electric Cell-Substrate Impedance Sensing (ECIS)

Trans-epithelial resistance (TER) of *C*. *muridarum* infected OE cell monolayers were measured with the ECIS system using the Zθ (Theta) model and using the 8W10E+ (8-well) arrays (Applied Biophysics Inc; Troy, NY). Prior to seeding the cells, the array chambers were treated with a 10mM solution of cysteine for 10 min at room temperature to stabilize the electrode. After removing the stabilization solution, the wells were washed twice with sterile distilled water and seeded with either the OE-129WT cells or the OE-TLR3(-) cells at a density of 10^5^ cells/cm^2^ in 400ml total volume epithelial media to achieve rapid confluence. After brief centrifugation for 10 min at 1000 x *g* to attach cells to the surface, the cells were incubated at 37°C in a CO_2_ incubator for 4hrs to become completely confluent, which was verified by microscopy before proceeding to the next step. After 4hrs of incubation, IFN-β was added to specific wells containing OE-TLR3(-) cells to achieve a final concentration of 50U/ml and returned to the CO_2_ incubator for an additional 1hr. After the 1hr incubation with IFN-β, all cells were infected with a multiplicity of infection (MOI) of 1 IFU/cell using the method described above. Resistance, capacitance, and impedance information were collected continuously for 48hrs post-infection. Each experiment had two replicate wells per array, and the experiment was repeated at least six times.

### Transwell cell permeability assays

OE-129WT and OE-TLR3(-) cells were seeded at a density of 10^5^ cells/cm^2^ on the TC-coated polyester membrane inserts from the Corning Transwell 24-well plate system (Costar; Brumath, France). Mock treatment, IFN-β pre-treatment, and infection with an MOI of 1 IFU/cell *C*. *muridarum* were all performed as described above after cells had been growing in the CO_2_ incubator for 6hrs after seeding. FITC-dextran (molecular mass of 70kDa; Sigma) was used as an index of macromolecular permeabilization: Briefly, FITC-dextran was added to epithelial media to a final concentration of 1mg/mL and added to the upper chambers of the Transwell system. The lower chamber contained 300μl epithelial media, and 150μL samples were taken from the lower chamber at 6-hour intervals to measure the amount of FITC-dextran that traversed the monolayer during that time interval. The same volume was immediately replenished in the lower chamber with fresh epithelial medium to prevent fluid movement caused by hydrostatic pressure. The fluorescence was measured with a BioTek FLx800 fluorimeter (BioTek; Winooski, VT) using 480nm and 520nm as the excitation and emission wavelengths, respectively.

### RNA purification and quantitative real-time polymerase chain reaction (qPCR)

OE129-WT cells, OE-TLR3(-) cells, and OE-TLR3(-) cells that pre-treated with 50U/ml recombinant IFN-β were grown to confluence before being either mock-infected or infected with 10 IFU/ cell *C*. *muridarum*. The OE-TLR3(-) cells pre-treated with IFN-β had the 50U/ ml IFN-β added to the media 1hr before infection as described in [8]. Cell lysates were harvested at either 0, 8, 12, 20, or 36hr post-infection, and total cell RNA was isolated using the RNeasy plus kit (Qiagen; Valencia, CA) according to manufacturer’s protocol. The DNA-free RNA samples were quantified using the NanoDrop spectrophotometer (Thermo Scientific), and cDNA was obtained with the Applied Biosystem’s high-capacity cDNA reverse transcription kit (Thermo Fisher) using 500ng total cell RNA. Target gene cDNA was amplified using Applied Biosystem’s TaqMan gene-expression master kit in reactions containing primers for the various tight-junction (TJ) genes and/or the β-actin control primers (Table 1) according to manufacturer’s protocol. Quantitative measurements were performed via ABI7500 real-time PCR detection system (Thermo Fisher). Relative expression levels were measured as a fold increase in mRNA expression versus mock controls and calculated using the formula 2^−ΔΔCt^ as described in [33].

**Table 1:** Primers for qPCR.

	Sense Primer	Antisense Primer
CLDN1	*5’-AGGAGCAGGAAAGTAGGACA-3’*	*5’-GTCCCCCAACTTGAGATGTATG-3’*
CLDN2	*5’-AGCTGACTTCTTTCCTCCTTAC-3’*	*5’-TCGCCTTTCTCTGGACCTA-3’*
CLDN3	*5’-GTCTGTCCTCTTCTAGCCTA-3’*	*5’-CACTACCAGCAGTCGATGAAC-3’*
CLDN4	*5’-CACTCAGCACACCATGACTT-3’*	*5’-CACTCAGCCTACACGTTACTC-3’*
ZO-1	*5’-GCCACTACAGTATGACCATCC-3’*	*5’-AATGAATAATATCAGCACCATGCC-3’*
OCLDN	*5’-GTTGATCTGAAGTGATAGGTGGA-3’*	*5’-CACTATGAAACAGACTACACGACA-3’*
JAM-1	*5’-ACGAGGTCTGTTTGAATTCCC-3’*	*5’-GCCTATAGCCGTGGATACTTTG-3’*
β-actin	*5’-AGGTCTTTACGGATGTCAACG-3’*	*5’-ATTGGCAACGAGCGGTT-3’*

### SDS PAGE and Western blotting

OE-129WT cells, OE-TLR3(-) cells, and OE-TLR3(-) cells that pre-treated with 50U/ml recombinant IFN-β were grown to confluence before being either mock-infected or infected with 10 IFU/ cell *C*. *muridarum*. Cell lysates were harvested at either 0, 8, 12, 20, or 36hr post-infection, and were analyzed for tight-junction protein expression by SDS-PAGE and subsequent Western blotting techniques as described previously [32]. In brief, equal protein amounts were separated on 4–12% Tris-Glycine Gel (Novex, Invitrogen Carlsbad CA, USA) and blotted onto PVDF membrane (Amersham Hybond, GE Healthcare Life Sciences; Chicago, Illinois, USA). After blocking, PVDF membranes were immunoblotted with primary antibodies: rabbit anti-mouse claudin-1 (1:200 dilution; catalog# 51–9000), rabbit anti-mouse claudin-2 (1:100 dilution; catalog# 51–6100), rabbit anti-mouse claudin-3 (1:50 dilution; catalog# PA5-16867), mouse anti-mouse claudin-4 (1:50 dilution; catalog# 32–9400), rabbit anti-mouse ZO-1(1:2000 dilution; catalog# 61–7300) and goat anti-mouse JAM1 (1:5000 dilution; catalog# PA5-47059), all purchased from Invitrogen. Rabbit anti-mouse occludin (1:250 dilution; catalog# LS-B2187) was purchased from LsBio (LifeSpan BioSciences Inc., Seattle, WA) and the monoclonal antibody specific for mouse β-actin (1:50000 dilution; catalog# A1978) antibody was purchased from Sigma. The HRP conjugated goat anti-rabbit IgG (catalog# 32460), and goat anti-mouse IgG (catalog# 32430) were purchased from Thermo Scientific, while the chicken anti-goat IgG (catalog# CkxGt-003-EHRPX) was purchased from ImmunoReagent Inc. (Raleigh, NC). All HRP-conjugated antibodies were diluted to 1:7500 dilution and served as secondary antibodies in this study. Detection of specific proteins was done using the Super-Signal West-Dura extended duration substrate (Thermo Scientific) and was carried out according to the manufacturer’s protocol. For quantification of western blots, western blot images were analyzed with NIH’s ImageJ software according to the tutorial described on the website: http://lukemiller.org/index.php/2010/11/analyzing-gels-and-western-blots-with-image-j/. Statistical analysis on the obtained density values was carried out with Microsoft Excel and the GraphPad Prism program. The relative band intensity of each tight-junction protein was obtained by the normalization to the band intensity of the β-actin loading control. Experiments were repeated at least three times.

### Immunofluorescent staining of tight junction proteins

OE-129WT cells and OE-TLR3(-) cells were grown to confluence in 96-well Ibidi plates (Ibidi USA; Fitchburg, Wisconsin) before being either mock-infected or infected with 1 IFU/ cell *C*. *muridarum* at 37°C for up to 36hrs. At the specified time-point, the cells were carefully rinsed with PBS before being fixed 10 min at room temperature using 10% neutral-buffered formalin (Sigma). The monolayers were washed at room temperature 3 times for 5 min using PBS. After washing in PBS, the cells were blocked and permeabilized for 30 min using PBS containing 1% BSA and 0.1% saponin. After permeabilization, cells were washed in blocking buffer (PBS + 1% BSA) at room temperature for 5 min, and the primary antibody (either Claudin-1; 1:50 dilution, ZO-1; 1:100 dilution, or JAM-1; 1:200 dilution) was added to the cells for 1hr at room temperature. After washing at room temperature 3 times for 5 min using PBS, the cells were then incubated in the dark at room temperature for 1hr using either goat anti-rabbit, goat anti-mouse, or donkey anti-goat 2° antibody conjugated with Alexa Fluor-594 (1:1000 dilution, Molecular Probes). Finally, the cells were washed 3 times for 5 min using PBS and counterstained with DAPI to identify the nuclear DNA. Duplicates processed without primary antibodies served as negative controls. Fluorescence was imaged using a Leica DMI 6000B inverted fluorescent microscope.

### Statistical analysis

Numerical data are presented as mean ± (SD). All experiments were repeated at least three times, and statistical significance was determined using Student’s two-tailed *t*-test unless stated otherwise. 2-way *ANOVA* analyses in GraphPad Prism were used to analyze the differences in IFUs recovered from mouse upper genital tract tissue homogenates. Statistically significant differences are shown by asterisks (*), and with the minimum criteria being *p* <0.05.

## Results

### *Chlamydia muridarum* ascends more rapidly into the upper genital tract tissues of TLR3-deficient mice

**Fig 1:**
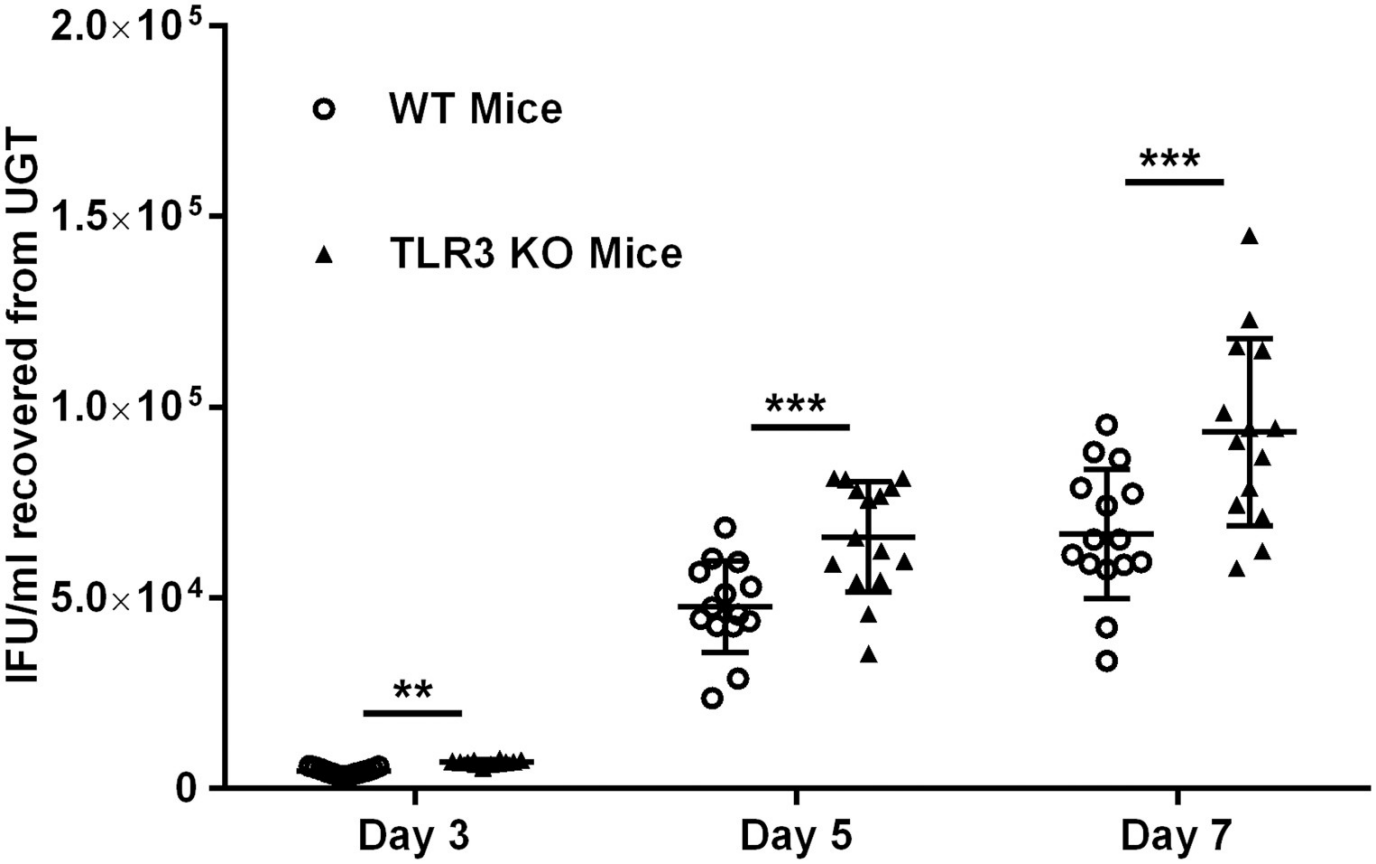
*C*. *muridarum* recovery from upper genital tract (UGT) tissue of wild-type and TLR3-KO mice. Genital tract infections were performed, and UGT tissue was collected on days 3, 5, and 7 post-infection as described in Materials and Methods. The live chlamydial organisms recovered from the UGT were titrated, and the results were expressed as IFUs/ml recovered along the Y-axis. Data shown are pooled results of 3 individual experiments resulting in the total of *n* = 15 mice for each strain on days 3, 5, and 7. IFU = inclusion forming units. The data are represented as mean (SD) ** = *p <0*.*005 and* *** = *p <0*.*001 when comparing WT vs*. *TLR3-deficient mice on that given day*.

We previously showed that TLR3-deficient mice exhibited significantly higher levels of chlamydial shedding from the lower genital tract when compared to wild-type (WT) mice during the first four weeks of infection [6, 27]. Additionally, our earlier *in vitro* studies showed that *Chlamydia* replication in oviduct epithelial (OE) cells deficient in TLR3 was more robust than in the wild-type OE cells [8]. The data from those previous experiments showed that TLR3 had a biological impact on the innate immune response to *Chlamydia* infection in mice, and we hypothesized that TLR3 deficiency might have a subsequent impact on *Chlamydia’s* ability to spread and ascend the female genital tract. To ascertain whether TLR3’s function indeed has a role in the chlamydial spread and ascension into the upper genital tract (UGT) tissue of female mice, we infected WT and TLR3-deficient (TLR3-KO) mice with 10^5^ IFU *C*. *muridarum* and harvested the UGT tissue from mice at either day 3, 5, or 7 post-infection. As shown in Fig 1, infectious chlamydial elementary bodies (EBs) were recovered from the UGT of the infected mice, thus demonstrating that *C*. *muridarum* reaches the tissue encompassing the oviduct and uterine horn as early as 3 days post-infection. We consistently recovered significantly more infectious chlamydial progeny from TLR3-deficient mice at all time points tested in each experiment. These findings are consistent with our previous reports of more robust *Chlamydia* replication in the absence of TLR3 function [6, 8, 27]. The observation that there was significantly more chlamydial progeny in the UGT tissues of mice early during infection supports our hypothesis that TLR3 deficiency leads to a more progressive infection, increases chlamydial spread, and demonstrates a more rapid ascension of *Chlamydia* into the upper reproductive tract tissues.

### TLR3 deficiency augments the chlamydial degradation of OE cell TER

**Fig 2:**
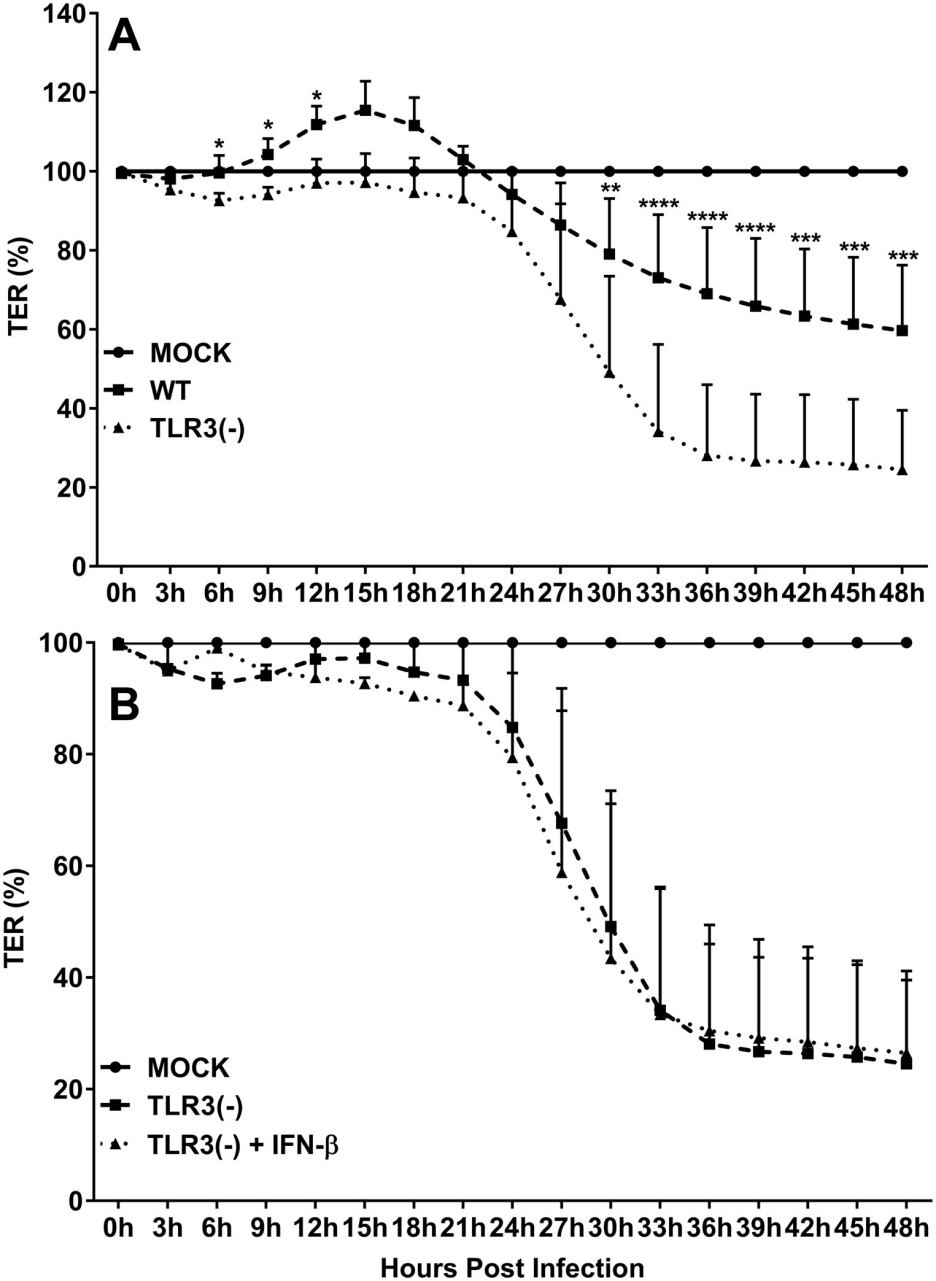
TLR3 deficiency exacerbates the *Chlamydia*-induced attenuation of trans-epithelial resistance (TER) in murine OE cell monolayers. TER was measured every three hours in mock and *C*. *muridarum* infected: **A)** WT vs. TLR3-deficient OE cells, and **B)** TLR3-deficient OE cells vs. TLR3-deficient OE cells pre-treated with 50U/ml IFN-β 1hr prior to infection. TER at each time-point is relative to mock-infected controls of each respective cell line set at 100%. Data are representative of 6 independent experiments. WT = wild-type and TLR3(-) = TLR3-deficient OE cells. The data are represented as mean (SD). *Two-way ANOVA with Tukey’s multiple comparisons test applied*. *P <0.*05*, ** = *p ≤0*.*0025*, *** = *p 0*.*0003 thru 0*.*0001*, **** = *p <0*.*0001 comparing WT and TLR3-deficient OE cells at the given time*.

It has been demonstrated in other studies that *Chlamydia* infection can impact epithelial barrier function by inducing the secretion of various inflammatory mediators that are hypothesized to modulate the synthesis of host proteins that form cellular tight junctions (TJs) and adherens junctions (AJs) [34–37]. To determine if TLR3 deficiency has any impact on *Chlamydia’s* ability to affect host cell barrier integrity, we infected wild-type and TLR3-deficient OE cells with 1 IFU/ cell *C*. *muridarum* and measured the trans-epithelial resistance (TER) of the cell monolayers over a period of 48hrs in ECIS. As shown in Fig 2A, the TER in wild-type OE cells is significantly increased in response to *Chlamydia* infection after 6hrs post-infection when compared to infected TLR3-deficient OE cells and the mock controls. However, the TER begins to precipitously decline after about 24hrs (40% reduction in TER in the wild-type OE cells at the 48hr time point, *p< 0*.*05*). These data are in accordance with previous studies showing a reduction in TER at a late time point during *Chlamydia* infection [34]. However, we did not observe an initial increase in TER in the TLR3-deficient OE cells as we had seen in the wild-type OE cells, which corroborates reports by others suggesting a critical role for TLR3 in increasing epithelial barrier function early during the epithelial repair process [38, 39]. The rate of degradation in TER was significantly higher in the TLR3-deficient OE cells, which resulted in an almost 80% decline in TER by 36hrs post infection. We previously reported that *C*. *muridarum*-induced synthesis of IFN-β in TLR3-deficient OE cells was significantly reduced and that *Chlamydia* replication in the absence of TLR3-induced IFN-β was significantly increased in those cells when compared to wild-type OE cells [6]. Pretreatment of TLR3-deficient OE cells with 50U/ml exogenous IFN-β for 1hr before infection resulted in a significant reduction chlamydial progeny; however, pre-treatment with exogenous IFN-β had no significant impact on *C*. *muridarum* infection and replication in wild-type OE cells [8]. To examine whether IFN-β has an impact on the more rapid decline of TER during TLR3 deficiency, we pretreated TLR3-deficient OE cells with 50U/ml of exogenous IFN-β 1hr prior to infection; however, the TLR3-deficient OE cells exhibited no significant change TER during infection when compared to the untreated TLR3-deficient OE cells (Fig 2B). Also, we did not see any significant difference in the TER of WT OE cell monolayers that were pre-treated with 50U/ml recombinant IFN-β for 1hr before infection (S1 Fig), indicating that IFN-β had no impact on the TER during *C*. *muridarum* infection in either OE cell type. The ECIS data show that TLR3 deficiency in oviduct epithelium led to a more rapid decline in TER during *Chlamydia* infection, and the rate of TER decline was not affected by the addition of exogenous IFN-β which is known to restrict *C*. *muridarum* replication in these cells.

### TLR3 deficiency exacerbates the *Chlamydia* infection induced macromolecular permeability in OE cell monolayers

**Fig 3:**
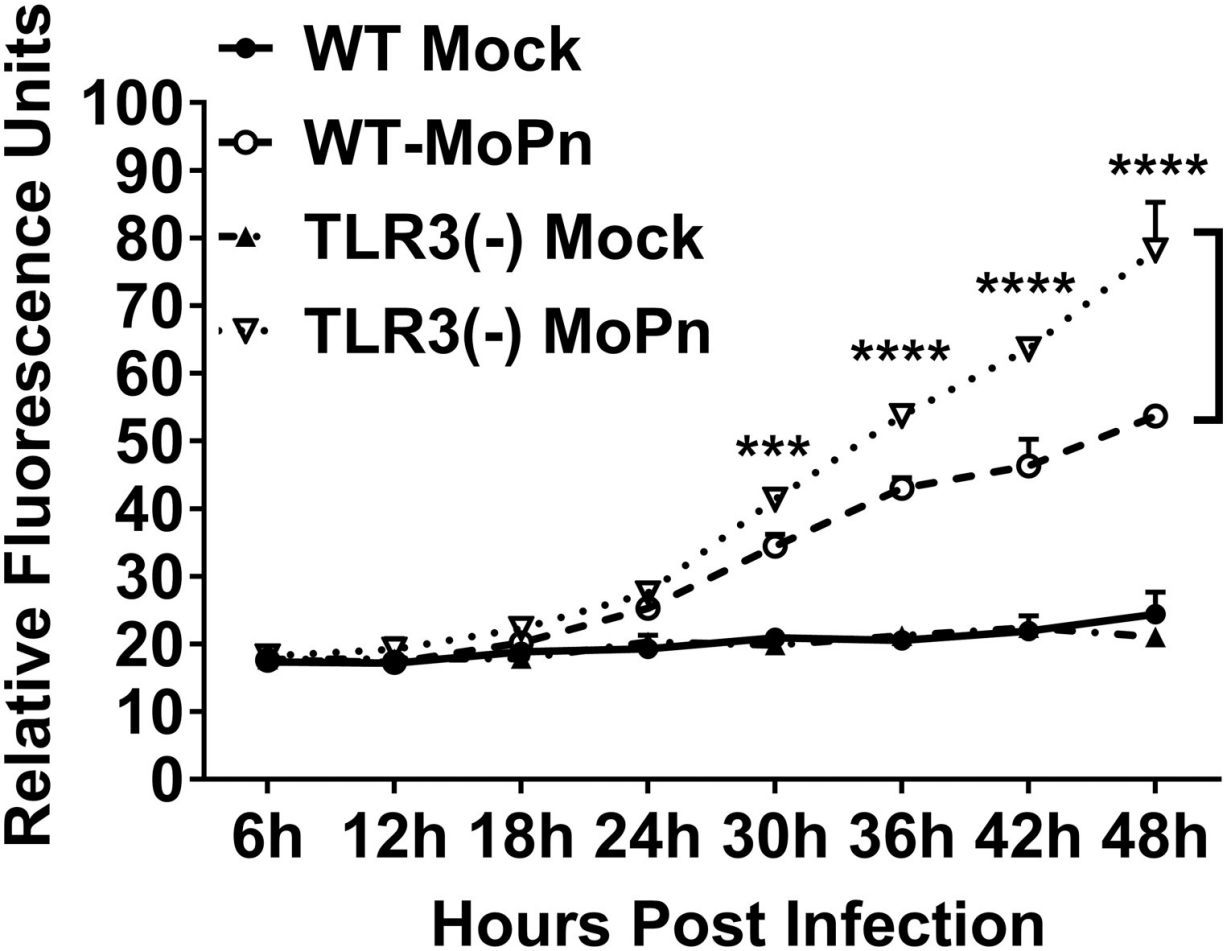
TLR3 deficiency leads increased rates of macromolecular permeability in OE cell monolayers during *Chlamydia* infection. Macromolecular permeability assays were performed in WT and TLR3-deficient OE cell lines that were either mock-infected or *C*. *muridarum*-infected at an MOI of 1 IFU/ cell. Relative permeability was measured using a FITC-labeled dextran (70-kDa) probe. Samples were taken from the basolateral chamber of the transwell every 6hrs post-infection, and permeability was determined by increases in relative fluorescence compared to mock-infected controls. Data are representative of three independent experiments. WT = wild-type and TLR3(-) = TLR3-deficient OE cells. The data are represented as mean (SD). *Two-way ANOVA with Tukey’s multiple comparisons test applied*. *** = *p 0*.*0003 thru 0*.*0001*, **** = *p <0*.*0001 comparing WT and TLR3-deficient OE cells at the given time*.

We next performed transwell macromolecular permeability assays to probe the physiological relevance of the pronounced decline of TER in TLR3-deficient OE cells during *C*. *muridarum* infection. Wild-type and TLR3-deficient OE cells were either mock-infected or infected with 1 IFU/ cell *C*. *muridarum* to determine if the drop in TER coincided with increased permeability of the OE cell monolayers to 70kDa FITC-dextran. As shown in Fig 3, *C*. *muridarum* infection significantly increased the amount of 70kDa FITC-dextran allowed to pass through the OE cell monolayers when compared to the mock-infected controls after about 18hrs post-infection in both cell types. However, the amount of FITC-dextran that traversed the TLR3-deficient OE cell monolayer was significantly higher than in wild-type cells after the 24hr time-point *(P< 0*.*05; denoted by an asterisk)*. We did not see any significant difference in the macromolecular permeability of either the WT or TLR3-deficient OE cell monolayer when the cells were pre-treated with 50U/ml recombinant IFN-β for 1hr before infection (S2 Fig), indicating that IFN-β had little impact on the macromolecular permeability of TLR3-deficient OE cells during *C*. *muridarum* infection. The macromolecular permeability results corroborate the ECIS data demonstrating that TLR3 deficiency leads to more dramatic reductions in TER in murine OE cells. Collectively, the ECIS and transwell experiments indicate that TLR3-deficient OE cells are more susceptible to *Chlamydia* infection-induced breakdown of the monolayer integrity, and suggest that TLR3 signaling plays a substantial role in the maintenance of epithelial barrier function in oviduct epithelium during *Chlamydia* infection.

### The transcription of tight junction (TJ) genes are differentially regulated in TLR3-deficient OE cells during *Chlamydia* infection

**Fig 4:**
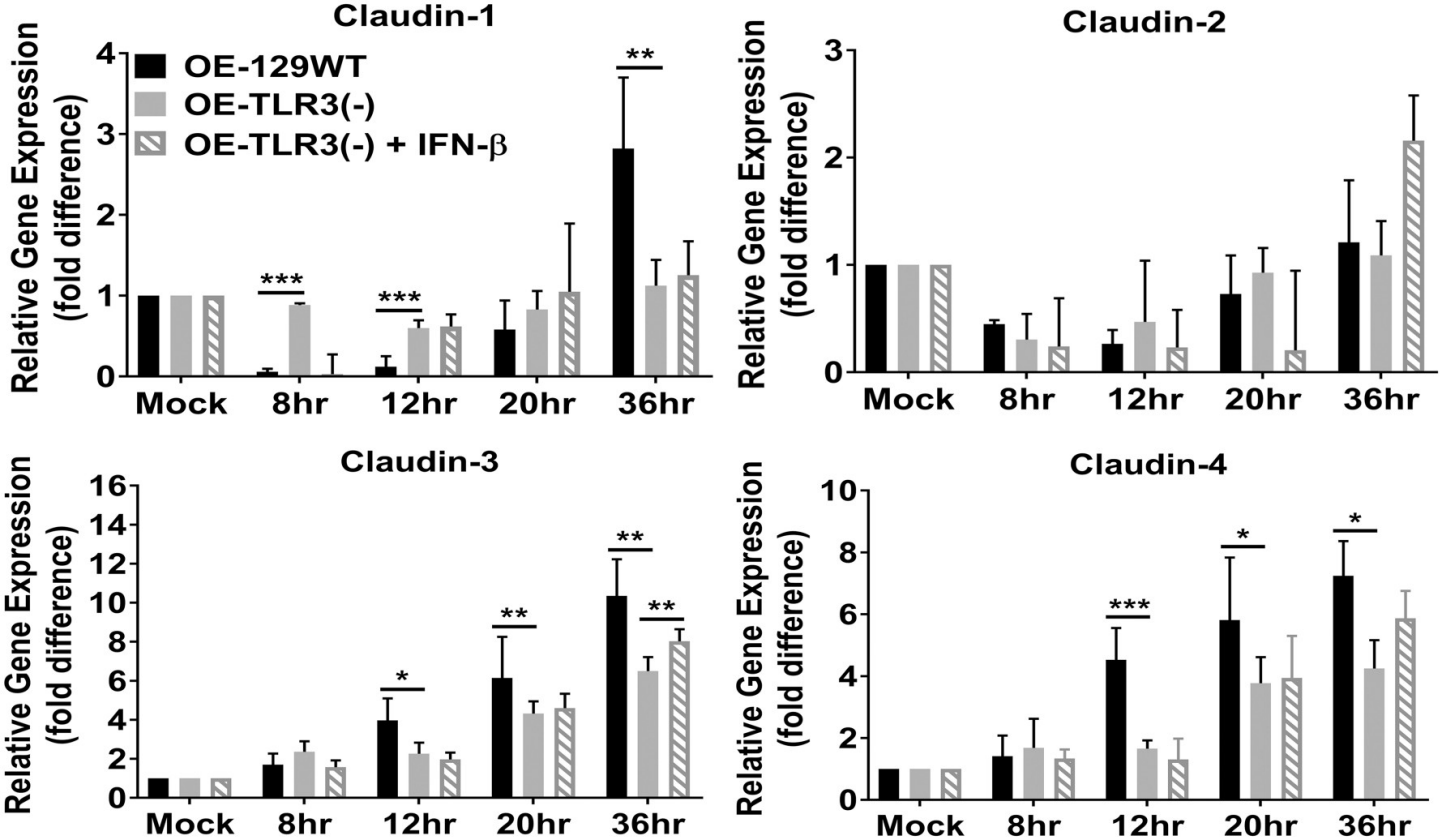
TLR3 deficiency dysregulates the *Chlamydia*-induced gene expression of the claudin integral membrane tight junction (TJ) proteins. Gene expression levels of Claudins 1–4 were measured by qPCR at various times post-infection in *C*. *muridarum* infected WT, TLR3-deficient, and TLR3-deficient OE cells that were pre-treated with 50U/ml recombinant IFN-β 1hr before infection. Data are representative of three or more independent experiments. OE-129WT = wild-type and OE-TLR3(-) = TLR3-deficient OE cells. The data are represented as mean (SD). * = *p <0*.*05*, ** = *p <0*.*005*, and *** = p <0.*001 when compared to TLR3-deficient OE cells at the given time*.

The more dramatic reduction in TER appears to coincide with increased macromolecular permeability in the TLR3-deficient OE cells late during *C*. *muridarum* infection. These results imply that TLR3-deficiency leads to a more acute breakdown in the integrity cellular tight junctions (TJs) and/or adherence junctions (AJs) that are known to be affected during cellular invasion by specific viral and bacterial pathogens [34, 36, 37, 40–42]. To ascertain whether the dissimilarity in macromolecular permeability and TER between the wild-type and TLR3-deficient OE cells correlates with a differential regulation in TJ gene expression during *Chlamydia* infection, we measured gene transcription levels of the candidate TJ proteins by quantitative real-time-PCR (qPCR). OE129-WT, OE-TLR3(-), and OE-TLR3(-) cells pre-treated with IFN-β were either mock-infected or infected with 10 IFU/ cell *C*. *muridarum* before being harvested for total RNA isolation at 0, 8, 12, 20, and 36hrs post-infection. As shown in Fig 4, claudin-1 was significantly downregulated early (0-8h) and mid-infection (9-18h) in the OE129-WT cells, but eventually recovered to the levels of the mock-infected controls by 20hrs post-infection. However, claudin-1 gene transcription levels were significantly higher in the OE129-WT cells when compared to the mock-infected control cells by the 36hr time-point. In contrast, claudin-1 transcription was not as affected early during infection in the OE-TLR3(-) cells, and its expression levels were 3-fold lower than the OE129-WT cells at the 36hr time-point. Claudin-2 transcription was not very different between the two cell types, and its expression was also slightly downregulated early and mid-infection in both OE cell types. Claudins 3 and 4 gene expression levels were upregulated in both cell types; however, their expression levels were significantly reduced in the OE-TLR3(-) cells at most times past the 12hr time-point. There was no significant impact on transcription of any of the claudin genes tested when IFN-β was added to OE-TLR3(-) cells 1hr before infection, except for the 36hr timepoint when claudin-3 transcription was significantly higher in the IFN-β pre-treated TLR3-deficient OE cells.

**Fig 5:**
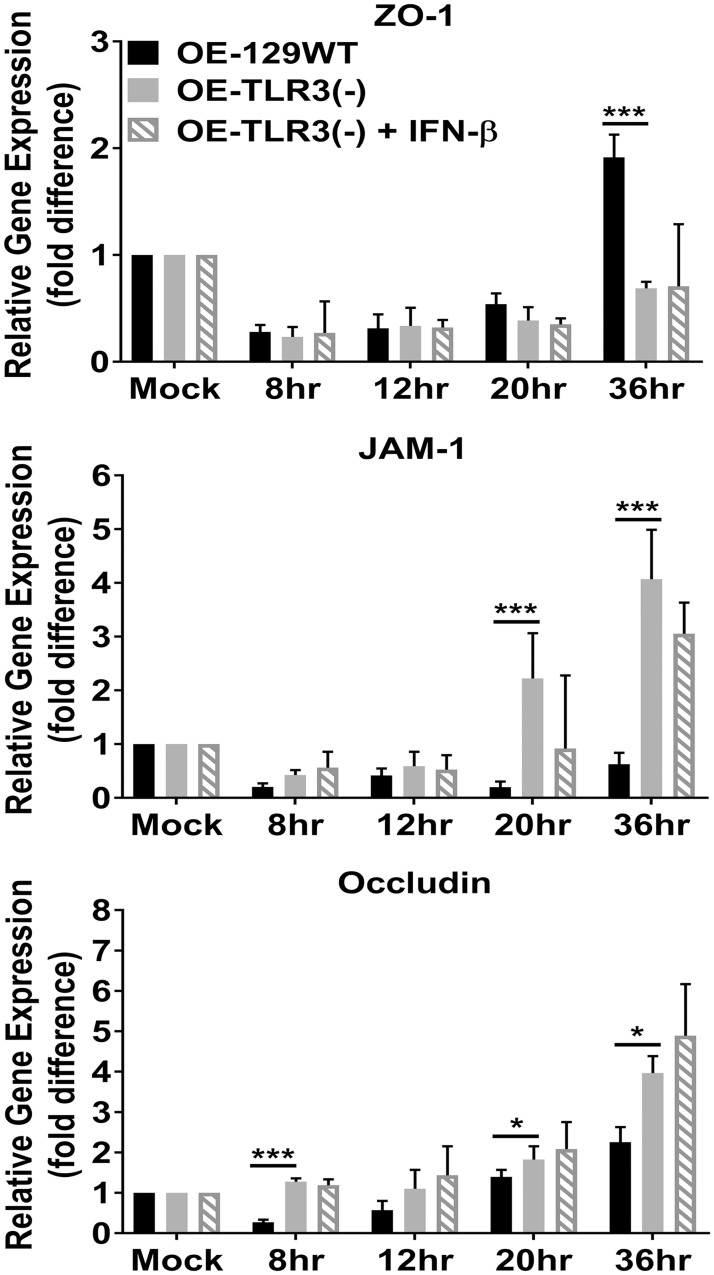
The *Chlamydia*-induced gene expression of the TJ proteins is impacted during TLR3 deficiency. Gene expression levels of ZO-1, JAM-1, and occludin were measured by qPCR at various times post-infection in *C*. *muridarum* infected WT, TLR3-deficient, and in TLR3-deficient OE cells that were pre-treated with 50U/ml recombinant IFN-β 1hr before infection. Data are representative of three or more independent experiments. OE-129WT = wild-type and OE-TLR3(-) = TLR3-deficient OE cells. The data are represented as mean (SD). * = *p <0*.*05 and* *** = *p <0*.*001 when comparing WT vs*. *TLR3-deficient OE cells at the given time*.

Fig 5 shows qPCR results of the *Chlamydia*-induced gene expression of ZO- 1, JAM-1, and occludin in the OE129-WT, OE-TLR3(-), and IFN-β treated OE-TLR3(-) cells. As indicated, ZO-1 transcription levels were evenly downregulated during *C*. *muridarum* infection in all cell types, but gene expression levels began to rise in the OE129-WT cells starting at 20hrs post-infection. By the 36hr time-point, ZO-1 gene expression levels were more than 2-fold higher in the OE129-WT cells when compared to the OE-TLR3(-) cells. In contrast to ZO-1 results, we saw an initial uneven downregulation in JAM-1 transcription, but it was the OE-TLR3(-) cells that had a dramatic increase in JAM-1 gene expression starting at 20hrs post-infection. By the 36hr time-point, the JAM-1 gene expression was almost 8-fold higher in the OE-TLR3(-) cells when compared to the OE-129WT cells.

Occludin transcription was significantly downregulated early during infection of the OE-129WT cells but slowly increased to a little over a 2-fold difference in gene expression levels versus the mock-infected cells at the 36hr time-point. In contrast, occludin transcription levels remained relatively stable in the OE-TLR3(-) cells before increasing to almost 4-fold versus the mock-infected controls at the 36hr time-point. Again, we saw no significant differences between OE-TLR3(-) cells and IFN-β treated OE-TLR3(-) cells. However, we did notice that the TJ gene expression levels trended towards the direction of their respective counterpart in OE-129WT cells in all cases except for occludin, where it trended more towards the OE-TLR3(-) cell results. Because OE-129WT cells secrete significantly higher amounts of *C*. *muridarum*-induced IFN-β starting as early as 2-3hr post-infection [43], we saw no significant differences in the candidate TJ protein transcription levels when the OE-129WT cells were pre-treated with 50U/ml of exogenous IFN-β (S3 and S4 Figs). Collectively, our data show that TJ gene expression levels in OE cells are affected by *Chlamydia* infection, which supports the findings of other investigators using different cell types [34, 35]. However, we show that the *Chlamydia’s* impact on TJ gene expression in OE cells is differentially regulated during TLR3 deficiency.

### TLR3 signaling has a differential impact on the synthesis, stability, and cellular distribution of candidate TJ proteins during *Chlamydia* infection of OE cells

**Fig 6:**
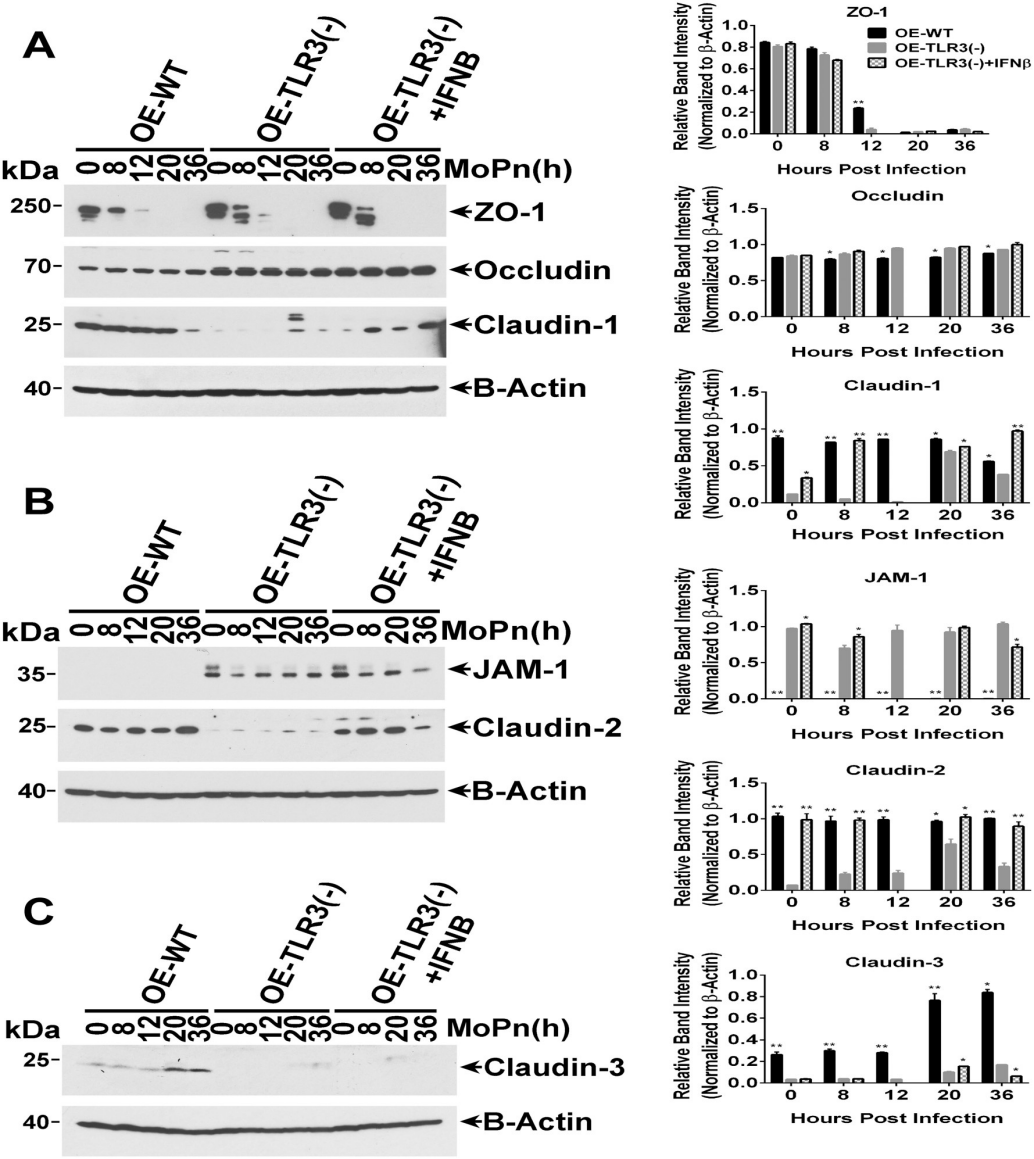
TLR3 deficiency leads to altered TJ protein expression in OE cell monolayers during *Chlamydia* infection. WT, TLR3-deficient, and IFN-β pre-treated TLR3-deficient OE cells were infected with 10 IFU/ cell *C*. *muridarum* for up to 36 hours before cell lysates were harvested at the various time-points shown. Protein expression levels were measured by western blot analyses for: **(A)** ZO-1, occludin, and claudin-1; **(B)** JAM1 and claudin-2; and **(C)** claudin-3. OE-129WT = wild-type and OE-TLR3(-) = TLR3-deficient OE cells. *Blots shown are representative data*. **Right-side plots** show densitometry of tight-junction proteins normalized to the β-actin control for the representative data. The relative band intensity is the ratio number of respective band intensity for each TJ protein (minus background) at the indicated time-point, divided by the band intensity for β-actin (minus background) at that specific time-point shown in the *representative western blot data*. Significance was determined by comparing the ratio number of the representative data for WT and IFN-β pre-treated TLR3-deficient OE cells, to the ratio number of OE-TLR3(-) cells divided by β-actin for three independent experiments. P-values were calculated using a two-tailed unpaired *t*-test, error bars represent mean (SD) * = *p <0*.*05 and* ** = *p <0*.*005* when compared to TLR3-deficient OE cells at the given time.

We next sought to determine if the *Chlamydia*-induced changes in the mRNA expression levels of candidate TJ genes translate into corresponding changes in their protein expression levels and/or stability. As shown in Fig 6A, ZO-1 protein expression levels in OE-129WT cells was rapidly diminished by 8hrs post-infection, was completely gone by 20hrs post-infection, but that there was little evidence of protein degradation. In contrast, ZO-1 expression was also expressed in the OE-TLR3(-) cells, but appeared to be a lot less stable and showing more signs of protein degradation since there was no full-length ZO-1 protein at the 12hr time-point. Occludin protein expression level and stability in OE-129WT cells remained steady throughout infection. Occludin protein expression in the OE-TLR3(-) cells was significantly higher immediately after infection (8hr time-point) when compared the OE-129WT cells but slightly increased throughout the remainder of infection. The stability of the occludin protein in the OE-TLR3(-) cells was mostly unaffected by the *Chlamydia* infection as it was in the OE-129WT cells, and pre-treating the OE-TLR3(-) cells with IFN-β prior to infection had only a minor impact on the stability and expression levels of both occludin and ZO-1.

The protein expression levels of claudins 1–3 were dramatically different between the OE-129WT cells and the OE-TLR3(-) cells (Fig 6A–6C). As shown, claudin-2 and claudin-3 proteins were expressed in the OE-129WT cells, and their expression levels either remained steady or were increased late during infection, while claudin-1 protein expression appeared to be diminished after the 20hr time-point. In contrast, the protein expression levels of claudins 1–3 were much lower in the OE-TLR3(-) cells; nonetheless, their expression levels were also moderately impacted by the *C*. *muridarum* infection. Interestingly, pre-treatment of OE-TLR3(-) cells with IFN-β prior to infection appeared to mildly but significantly increase the synthesis and stability of claudin-3 at the 20hr time point, while substantially bolstering the synthesis and stability of claudin-1 and claudin-2 throughout infection. These findings suggest that the *Chlamydia*-induced IFN-β synthesized via TLR3-signalling pathways plays a role in regulating the protein synthesis of these genes, while also simultaneously enhancing their protein stability within the cell. In contrast to the claudin proteins, JAM-1 protein expression was substantially higher in the OE-TLR3(-) cells when compared to the OE-129WT cells where it was barely detectable on the immunoblot. Pretreating the OE-TLR3(-) cells with IFN-β before *C*. *muridarum* infection seemed to result in a differential synthesis of JAM-1 throughout *Chlamydia* infection when compared to untreated OE-TLR3(-) cells, suggesting that the IFN-β synthesized via TLR3-signalling pathways may play a role in regulating the protein expression levels of JAM-1.

**Fig 7:**
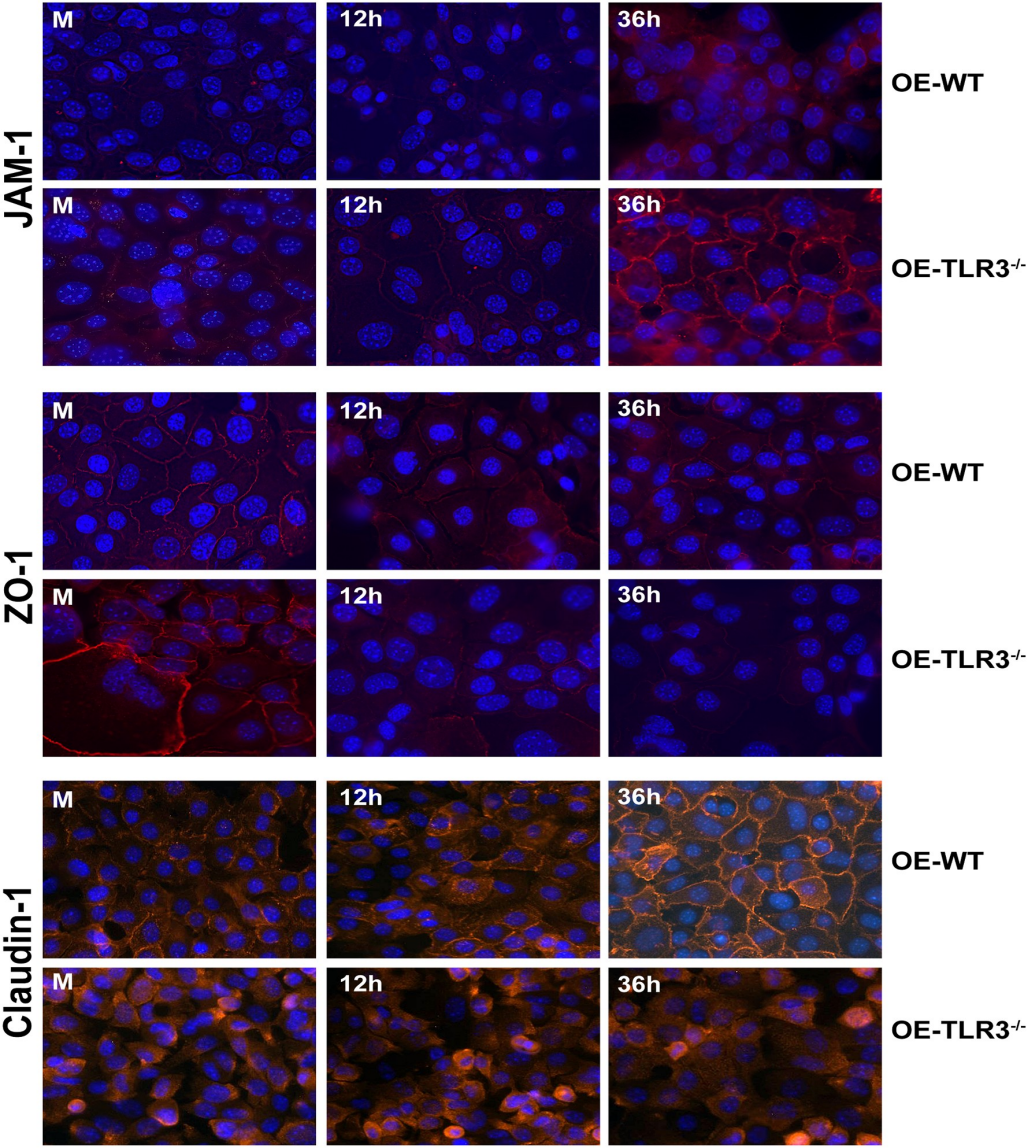
TLR3 deficiency impacts the chlamydial-induced rearrangement of claudin-1, JAM-1, and ZO-1 in cellular tight junctions. WT and TLR3-deficient OE cells were infected with a low MOI of 1 IFU/ cell *C*. *muridarum* for up to 36 hours before cells were fixed at the listed time-point post infection and examined by immunofluorescence as described in **Materials and Methods**. The intensity of the TJ fluorescence in the infected cells was differentially regulated throughout infection in the TLR3-deficient OE cells when compared to WT OE cells. OE-WT = wild-type and OE-TLR3(^-/-^) = TLR3-deficient OE cells. All images are 40x magnification and nuclei were counterstained with DAPI (blue).

Fluorescent microscopy was also performed to assess protein expression levels and cellular distribution of various TJ proteins in WT and TLR3-deficient OE cells over the course of *Chlamydia* infection. As shown in Fig 7, we observed a differential distribution of JAM-1, ZO-1, and claudin-1 to the cellular TJs throughout *Chlamydia* infection that seemed to depend on the functionality of TLR3. In corroboration with the western blot data, we saw substantially higher cellular expression levels of JAM-1 during *C*. *muridarum* infection in the TLR3-deficient OE cells, and its distribution along the outer membrane at cell-cell junctions increased at late times post-infection. In contrast, JAM-1 protein expression levels were noticeably lower in the OE-129WT cells and distribution at late times became increasingly cytoplasmic. ZO-1 protein expression levels along the outer membrane only slightly decreased by mid-infection in the OE-129WT cells, but its distribution remained mostly stable at cell-cell barriers late in infection even though its protein expression level had decreased. The expression and cellular distribution of ZO-1 differed during TLR3-deficiency in that its protein expression levels dramatically decreased over the course of infection, and its distribution became increasingly cytoplasmic in the TLR3-deficient OE cells late during *C*. *muridarum* infection. Claudin-1 protein expression levels remained mostly stable at mid-infection (12h) in the OE-129WT cells while only slightly increasing very late during infection; however, its distribution remained mostly stable at the cell-cell junctions throughout infection. Claudin-1 protein expression levels seemed to slightly diminish at late times post-infection in the TLR3-deficient OE cells, and its cellular distribution remained mostly cytoplasmic throughout infection. Results of the immunofluorescence studies reveal that *C*. *muridarum* infection can impact the cellular distribution of specific TJ proteins and likely the stability of cellular TJs of genital tract epithelium *in vitro*, which a finding that supports the investigation of others [34, 35, 44]. Additionally, the immunofluorescence data corroborate the western blot findings showing that TLR3 signaling plays a role in mitigating *Chlamydia’s* impact on TJ protein expression and its stability. Collectively, we demonstrate that TLR3 signaling plays a biological role in maintaining epithelial barrier function, and our data implicate *Chlamydia* infection, TLR3, and the IFN-β synthesized through TLR3 signaling pathways in the assembly and stability of cellular tight junctions during *Chlamydia* infection of OE cell monolayers.

## Discussion

Our previous reports proposed a protective role for TLR3 signaling in the immune response to *Chlamydia* infection in mice and suggested that TLR3 signaling triggers mechanisms that bolster the host’s ability limit the spread of *Chlamydia* and attenuate genital tract pathogenesis caused during infection. In this report, we investigated whether modulation of epithelial barrier function within the genital tract would be an example of such a mechanism where TLR3 signaling can exert its role in limiting *Chlamydia* spread and subsequent reproductive tract pathology. Our data showed that there were significantly higher amounts of *C*. *muridarum* in the UGTs of TLR3-deficient mice within the first seven days of infection when compared to wild-type controls. These findings corroborate our earlier reports showing that TLR3 deficiency in mice leads to higher chlamydial loads in the LGT, but we now show that these mice also suffer from a more rapid ascension of *C*. *muridarum* into the UGT.

The primary physical barrier in the female reproductive tract is the mucosal layer that lines the vagina, uterus, and luminal surfaces of the oviduct. The mucosal layer of the reproductive tract is an immunologically active layer that is comprised of an epithelial layer with tight cell-to-cell contact, which overlaid by a viscous layer of mucus [45]. The impact of *Chlamydia* infection on the mucosal barrier function has been previously investigated by others, and it was shown that *Chlamydia* infection disrupts the expression of critical proteins that form the cell-cell junctions (i.e., TJs and AJs) that play critical roles in forming intercellular adhesions [34–36, 46, 47]. The disruption of these intercellular adhesions during infection by *Chlamydia* is hypothesized to be an essential immune-evasion strategy that involves recognition of specific chlamydial PAMPs, which then triggers signaling events that manipulate host-cell structure and function to help facilitate its spread within the host [44, 46]. In addition to the chlamydial downregulation in the secretion of specific immune regulators and anti-microbial peptides, the chlamydial disruption of intercellular adhesions is also thought to play a significant role in increasing host vulnerability to co-infection with other genital tract pathogens such as gonorrhea and HIV-1 [34].

The identification of specific cellular mechanisms that are involved in the disruption and subsequent destabilization of epithelial barrier function during *Chlamydia* infection is a recent area of study in the pathogenesis of genital tract *Chlamydia* infections. The chlamydial induction of TLR-dependent inflammatory mediators such as IFN-γ, TNFα, and IL-1β has been shown to impact the gene expression level and the stability of TJ proteins and thereby inducing significant changes in TER when compared to uninfected controls [35, 48–52]. Because IFN-γ, TNFα, and IL-1β are all differentially expressed in the context of TLR3 deficiency in murine OE cells and mice [8, 27], we hypothesized that TLR3 deficiency might have a significant impact on the integrity of the epithelial barrier within the female reproductive tract during *Chlamydia* infection, due to the altered expression of these and other key cytokines.

We showed that *C*. *muridarum* has a significant impact on the deterioration of TER in OE cell monolayers during infection, and our findings support the investigations of others showing that *Chlamydia* infection causes significant changes in TER in other cell types when compared to uninfected controls [34, 35]. Also, we showed that there was a more rapid rate of decline in TER in the TLR3-deficient OE cells, which corresponded with a significantly increased rate in macromolecular permeability through the TLR3-deficient OE cell monolayers after 24hrs post-infection (see Fig 3). Collectively, our data indicate that TLR3 deficiency results in a more rapid breakdown in epithelial barrier function during *Chlamydia* infection, and proposes the hypothesis that *Chlamydia* exploits this phenomenon during TLR3 deficiency as a mechanism to accelerate its rate of ascent into the UGT of female mice early during infection (Fig 1) and causing increased pathology relative to WT mice [27].

However, other possible mechanisms can contribute to increased chlamydial replication and more rapid spread of *C*. *muridarum* into the UGTs of TLR3-deficient mice. For example, it was shown that dysregulation in the syntheses of IFN-γ, IFN-β, and other inflammatory factors that formulate the immunological barrier of the female reproductive tract could lead to increased tissue pathology and have a direct impact on augmenting chlamydial replication and enhancing its subsequent spread (reviewed in [53]); [8]). As a result, the increased chlamydial load enhances the host’s exposure to chlamydial virulence factors such as the chlamydial protease-like activity factor (CPAF), which is proteolytic protein known to play a role in fostering immune evasion by degrading transcription factors that are essential for regulating the expression of MHC molecules [54, 55]. CPAF is also known to directly enhance chlamydial viability by preventing the release of noninfectious RBs from infected cells via degradation of pro-apoptotic proteins, which inhibits cell death to facilitate completion of the chlamydial developmental cycle [56]. CPAF also plays a role in enhancing chlamydial viability by stabilizing the *Chlamydia* inclusion via Golgi-fragmentation as a mechanism to increase the transport of lipids into the chlamydial inclusion membrane [57]. Taken together, the more rapid ascension of *Chlamydia* into the UGTs of TLR3-deficient mice is likely the combined result of the dysregulation in syntheses of multiple cytokines and chemokines that formulate the immunological barrier in TLR3-deficient OE cells and mice [6, 8, 27], and a more rapid breakdown of the physical epithelial barrier in TLR3-deficient OE cells that functions to prevent the spread and transmission of sexually acquired infections [45, 58].

The exact link between TLR3 signaling and epithelial barrier function is unclear; however, our data showed that TLR3 deficient cells were differentially regulated in the expression of several candidate TJ genes in the *C*. *muridarum*-infected OE cells. Interestingly, our data showed that some TJ genes were upregulated while some were downregulated in response to *Chlamydia* infection and that some were also differentially regulated dependent upon the functionality of TLR3. The precise relationship between a particular TJ protein synthesis level and its specific impact on epithelial barrier function is unclear and requires further study. Functionally, TJs are known to play a major role in controlling paracellular permeability of the epithelial barrier, and the cellular pathways that modulate the individual TJ proteins are regulated by various host and pathogenic organism factors including cytokines, growth factors, virulence determinants from the invading pathogen, and hormones [59]. It is likely that the variability in syntheses and secretion levels of numerous cytokines, chemokines, and other innate-immune modulators during *Chlamydia* infection is the major contributing factor to explain why we observed both up and downregulation of the individual TJ genes.

The role of IFN-β in protecting the epithelial barrier was demonstrated in studies that showed that IFN-β significantly attenuated the IFN-γ-induced decrease in occludin and VE-cadherin expression in endothelial cells [60]. IFN-β has also been shown to be able to increase the stability of candidate TJ proteins and prevent the macromolecular permeability in brain endothelial cell monolayers that were removed from coculture with astrocytes or were treated with histamine [61]. However, whereas we did not observe any significant impact on TER, macromolecular permeability, or TJ transcription levels on a consistent basis when OE-TLR3(-) cells were pre-treated with IFN-β prior to infection, we did notice some augmented protein stability in claudin-1, claudin-2, and claudin-3 (to a lesser degree) in the IFN-β pre-treated OE-TLR3(-) cells (Fig 6). These findings indicate that the chlamydial induction of TLR3-dependent IFN-β during infection may play some role in the stabilization of the epithelial barrier, and proposes a possible mechanism of action that IFN-β uses to help limit the replication spread of *C*. *muridarum* as we have previously reported [8].

Finally, our data propose a role for TLR3 signaling in maintaining the integrity of epithelial barrier function during genital tract *Chlamydia* infection; albeit a function that we hypothesize is important in helping contain the bacterial spread, but one that eventually becomes overwhelmed during productive infections as the pathogen ascends into the UGT. We show that TLR3 deficiency leads to more rapid ascension into the UGT of infected mice, and we attribute the more rapid ascension to dysregulation in the *C*. *muridarum*-induced syntheses of critical immune modulators and the increased infection-induced disruption of cell-cell junctions. We are the first to report a role for TLR3 in the maintenance of barrier integrity in oviduct epithelium during genital tract chlamydial infections; however, our work here corroborates a prior study in which the investigators reported a role for TLR3 in normal skin barrier repair following UVB damage [38]. In that study, the authors report changes to TER, paracellular transport of fluorescein-labeled sodium, and TJ protein expression when human keratinocytes were exposed to the TLR3 agonist poly-IC. The poly-IC was used to simulate the induction of snRNAs that are hypothesized to trigger TLR3 responses during UVB damage [39]. Our studies also parallel in that the prevailing hypothesis in both of our investigations involve a putative mechanism whereby TLR3 elicits this barrier maintenance function via cytokines and chemokines that are triggered upon stimulation by its appropriate PAMP. The identification of the specific immune factor that affects each specific aspect of the epithelial barrier function are subjects of our future investigations into this phenomenon.

## Supporting information

S1 Fig Trans-epithelial resistance (TER) in murine wild-type OE cell monolayers. TER was measured every three hours in mock and *C*. *muridarum* infected: WT OE cells and WT OE cells pre-treated with 50U/ml IFN-β 1hr prior to infection. TER at each time-point is relative to Mock-infected controls of each respective OE cell condition set at 100%. Data are representative of 6 independent experiments. WT = wild-type OE cells.
(TIF)

S2 Fig Macromolecular permeability in WT and TLR3-deficient OE cell monolayers that were pre-treated with IFN-β prior to *Chlamydia* infection. Macromolecular permeability assays were performed in **(A)** TLR3-deficient and **(B)** WT OE cell lines that were either untreated or pre-treated with 50U/ml recombinant IFN-β 1hr before being either mock-infected or *C*. *muridarum*-infected at an MOI of 1 IFU/ cell. Relative permeability was measured using a FITC-labeled dextran (70-kDa) probe. Samples were taken from the basolateral chamber of the transwell every 6hrs post-infection, and permeability was determined by increases in relative fluorescence compared to mock-infected controls. Data are representative of three independent experiments. WT = wild-type and TLR3(-) = TLR3-deficient OE cells.
(TIF)

S3 Fig *Chlamydia*-induced gene expression of the claudin integral membrane tight junction (TJ) proteins. Gene expression levels of Claudins 1–4 were measured by qPCR at various times post-infection in *C*. *muridarum* infected WT OE cells that were either mock-treated or pre-treated with 50U/ml recombinant IFN-β 1hr before infection. Data are representative of three or more independent experiments. OE-129WT = wild-type OE cells.
(TIF)

S4 Fig *Chlamydia*-induced gene expression of tight junction (TJ) proteins. Gene expression levels of ZO-1, JAM-1, and occludin were measured by qPCR at various times post-infection in *C*. *muridarum* infected WT OE cells that were either mock-treated or pre-treated with 50U/ml recombinant IFN-β 1hr before infection. Data are representative of three or more independent experiments. OE-129WT = wild-type OE cells.
(TIF)

## References

[1] CDC. New CDC analysis shows steep and sustained increases in STDs in recent years. 2018 August 28. Report No.

[2] CDC. DSTDP Facts-Chlamydia in the U.S. Atlanta, Georgia: 2004 May, 2004. Report No.

[3] Darville T. Chlamydia spp In: Nataro JP, Blazer M. J., and Cunningham-Rundles S., editor. Persistent Bacterial Infections. Washington D.C.: American Society of Microbiology; 2000 p. 229–61.

[4] CDC. 2015 Sexually Transmitted Diseases Survey 2017. https://www.cdc.gov/std/stats15/chlamydia.htm.

[5] Darville T, O’Neill JM, Andrews CW Jr., Nagarajan UM, Stahl L, Ojcius DM. Toll-like receptor-2, but not Toll-like receptor-4, is essential for development of oviduct pathology in chlamydial genital tract infection. J Immunol. 2003;171(11):6187–97. . 14634135 10.4049/jimmunol.171.11.6187

[6] Derbigny WA, Johnson RM, Toomey KS, Ofner S, Jayarapu K. The Chlamydia muridarum-induced IFN-beta response is TLR3-dependent in murine oviduct epithelial cells. J Immunol. 2010;185(11):6689–97. 10.4049/jimmunol.1001548 . 20974982

[7] Derbigny WA, Kerr MS, Johnson RM. Pattern recognition molecules activated by Chlamydia muridarum infection of cloned murine oviduct epithelial cell lines. J Immunol. 2005;175(9):6065–75. . 16237102 10.4049/jimmunol.175.9.6065

[8] Derbigny WA, Shobe LR, Kamran JC, Toomey KS, Ofner S. Identifying a role for Toll-like receptor 3 in the innate immune response to Chlamydia muridarum infection in murine oviduct epithelial cells. Infect Immun. 2012;80(1):254–65. 10.1128/IAI.05549-11 22006569 PMC3255657

[9] Prantner D, Darville T, Nagarajan UM. Stimulator of IFN gene is critical for induction of IFN-beta during Chlamydia muridarum infection. J Immunol. 2010;184(5):2551–60. Epub 2010/01/29. 10.4049/jimmunol.0903704 20107183 PMC2863030

[10] Welter-Stahl L, Ojcius DM, Viala J, Girardin S, Liu W, Delarbre C, et al Stimulation of the cytosolic receptor for peptidoglycan, Nod1, by infection with Chlamydia trachomatis or Chlamydia muridarum. Cell Microbiol. 2006;8(6):1047–57. 10.1111/j.1462-5822.2006.00686.x . 16681844

[11] Koropatnick TA, Engle JT, Apicella MA, Stabb EV, Goldman WE, McFall-Ngai MJ. Microbial factor-mediated development in a host-bacterial mutualism. Science. 2004;306(5699):1186–8. Epub 2004/11/13. 10.1126/science.1102218 . 15539604

[12] Da Costa CU, Wantia N, Kirschning CJ, Busch DH, Rodriguez N, Wagner H, et al Heat shock protein 60 from Chlamydia pneumoniae elicits an unusual set of inflammatory responses via Toll-like receptor 2 and 4 in vivo. European journal of immunology. 2004;34(10):2874–84. 10.1002/eji.200425101 . 15368304

[13] Erridge C, Pridmore A, Eley A, Stewart J, Poxton IR. Lipopolysaccharides of Bacteroides fragilis, Chlamydia trachomatis and Pseudomonas aeruginosa signal via toll-like receptor 2. J Med Microbiol. 2004;53(Pt 8):735–40. 10.1099/jmm.0.45598-0 . 15272059

[14] Netea MG, Kullberg BJ, Galama JM, Stalenhoef AF, Dinarello CA, Van der Meer JW. Non-LPS components of Chlamydia pneumoniae stimulate cytokine production through Toll-like receptor 2-dependent pathways. European journal of immunology. 2002;32(4):1188–95. 10.1002/1521-4141(200204)32:4<1188::AID-IMMU1188>3.0.CO;2-A . 11932927

[15] Prebeck S, Kirschning C, Durr S, da Costa C, Donath B, Brand K, et al Predominant role of toll-like receptor 2 versus 4 in Chlamydia pneumoniae-induced activation of dendritic cells. J Immunol. 2001;167(6):3316–23. . 11544320 10.4049/jimmunol.167.6.3316

[16] Alexopoulou L, Holt AC, Medzhitov R, Flavell RA. Recognition of double-stranded RNA and activation of NF-kappaB by Toll-like receptor 3. Nature. 2001;413(6857):732–8. 10.1038/35099560 . 11607032

[17] Matsumoto M, Kikkawa S, Kohase M, Miyake K, Seya T. Establishment of a monoclonal antibody against human Toll-like receptor 3 that blocks double-stranded RNA-mediated signaling. Biochemical and biophysical research communications. 2002;293(5):1364–9. 10.1016/S0006-291X(02)00380-7 . 12054664

[18] Jiang W, Pisetsky DS. The induction of HMGB1 release from RAW 264.7 cells by transfected DNA. Mol Immunol. 2008;45(7):2038–44. Epub 2007/11/23. 10.1016/j.molimm.2007.10.019 . 18031817 PMC3724460

[19] Lien E, Zipris D. The role of Toll-like receptor pathways in the mechanism of type 1 diabetes. Curr Mol Med. 2009;9(1):52–68. Epub 2009/02/10. . 19199942 10.2174/156652409787314453

[20] Matsumoto M, Funami K, Tanabe M, Oshiumi H, Shingai M, Seto Y, et al Subcellular localization of Toll-like receptor 3 in human dendritic cells. J Immunol. 2003;171(6):3154–62. . 12960343 10.4049/jimmunol.171.6.3154

[21] Ebihara T, Shingai M, Matsumoto M, Wakita T, Seya T. Hepatitis C virus-infected hepatocytes extrinsically modulate dendritic cell maturation to activate T cells and natural killer cells. Hepatology. 2008;48(1):48–58. Epub 2008/06/10. 10.1002/hep.22337 . 18537195

[22] Gowen BB, Hoopes JD, Wong MH, Jung KH, Isakson KC, Alexopoulou L, et al TLR3 deletion limits mortality and disease severity due to Phlebovirus infection. J Immunol. 2006;177(9):6301–7. Epub 2006/10/24. . 17056560 10.4049/jimmunol.177.9.6301

[23] Guillot L, Le Goffic R, Bloch S, Escriou N, Akira S, Chignard M, et al Involvement of toll-like receptor 3 in the immune response of lung epithelial cells to double-stranded RNA and influenza A virus. The Journal of biological chemistry. 2005;280(7):5571–80. 10.1074/jbc.M410592200 . 15579900

[24] Muller U, Steinhoff U, Reis LF, Hemmi S, Pavlovic J, Zinkernagel RM, et al Functional role of type I and type II interferons in antiviral defense. Science. 1994;264(5167):1918–21. . 8009221 10.1126/science.8009221

[25] Rudd BD, Burstein E, Duckett CS, Li X, Lukacs NW. Differential role for TLR3 in respiratory syncytial virus-induced chemokine expression. Journal of virology. 2005;79(6):3350–7. 10.1128/JVI.79.6.3350-3357.2005 . 15731229 PMC1075725

[26] Weber F, Wagner V, Rasmussen SB, Hartmann R, Paludan SR. Double-stranded RNA is produced by positive-strand RNA viruses and DNA viruses but not in detectable amounts by negative-strand RNA viruses. Journal of virology. 2006;80(10):5059–64. Epub 2006/04/28. 10.1128/JVI.80.10.5059-5064.2006 16641297 PMC1472073

[27] Carrasco SE, Hu S, Imai DM, Kumar R, Sandusky GE, Yang XF, et al Toll-like receptor 3 (TLR3) promotes the resolution of Chlamydia muridarum genital tract infection in congenic C57BL/6N mice. PLoS One. 2018;13(4):e0195165 10.1371/journal.pone.0195165 29624589 PMC5889059

[28] Johnson RM. Murine oviduct epithelial cell cytokine responses to Chlamydia muridarum infection include interleukin-12-p70 secretion. Infect Immun. 2004;72(7):3951–60. 10.1128/IAI.72.7.3951-3960.2004 15213139 PMC427409

[29] Schachter J. Chlamydiae (Psittacosis-lymphogranuloma venereum-trachoma group). 3rd ed ed: American Society for Microbiology, Washington, D.C.; 1980.

[30] Caldwell HD, Kromhout J, Schachter J. Purification and partial characterization of the major outer membrane protein of Chlamydia trachomatis. Infect Immun. 1981;31(3):1161–76. 7228399 10.1128/iai.31.3.1161-1176.1981 PMC351439

[31] Dai J, Tang L, Chen J, Yu P, Chen Z, Zhong G. The p47phox deficiency significantly attenuates the pathogenicity of Chlamydia muridarum in the mouse oviduct but not uterine tissues. Microbes Infect. 2016;18(3):190–8. Epub 2015/12/10. 10.1016/j.micinf.2015.11.003 . 26645958

[32] Derbigny WA, Hong SC, Kerr MS, Temkit M, Johnson RM. Chlamydia muridarum infection elicits a beta interferon response in murine oviduct epithelial cells dependent on interferon regulatory factor 3 and TRIF. Infect Immun. 2007;75(3):1280–90. 10.1128/IAI.01525-06 17178782 PMC1828549

[33] Livak KJ, Schmittgen TD. Analysis of relative gene expression data using real-time quantitative PCR and the 2(-Delta Delta C(T)) Method. Methods. 2001;25(4):402–8. Epub 2002/02/16. 10.1006/meth.2001.1262 . 11846609

[34] Mukura LR, Hickey DK, Rodriguez-Garcia M, Fahey JV, Wira CR. Chlamydia trachomatis regulates innate immune barrier integrity and mediates cytokine and antimicrobial responses in human uterine ECC-1 epithelial cells. Am J Reprod Immunol. 2017;78(6). Epub 2017/09/19. 10.1111/aji.12764 . 28921726

[35] Sze Ho L, He Q, Chen J, Xu P, Ling Tsang L, Yu S, et al Interaction between endometrial epithelial cells and blood leucocytes promotes cytokine release and epithelial barrier function in response to Chlamydia trachomatis lipopolysaccharide stimulation. Cell Biol Int. 2010;34(9):951–8. Epub 2010/06/19. 10.1042/CBI20100303 . 20557292

[36] Sun J, Kintner J, Schoborg RV. The host adherens junction molecule nectin-1 is downregulated in Chlamydia trachomatis-infected genital epithelial cells. Microbiology. 2008;154(Pt 5):1290–9. Epub 2008/05/03. 10.1099/mic.0.2007/015164-0 . 18451037

[37] Sun J, Schoborg RV. The host adherens junction molecule nectin-1 is degraded by chlamydial protease-like activity factor (CPAF) in Chlamydia trachomatis-infected genital epithelial cells. Microbes Infect. 2009;11(1):12–9. Epub 2008/11/06. 10.1016/j.micinf.2008.10.001 . 18983929

[38] Borkowski AW, Kuo IH, Bernard JJ, Yoshida T, Williams MR, Hung NJ, et al Toll-like receptor 3 activation is required for normal skin barrier repair following UV damage. The Journal of investigative dermatology. 2015;135(2):569–78. Epub 2014/08/15. 10.1038/jid.2014.354 25118157 PMC4289479

[39] Borkowski AW, Park K, Uchida Y, Gallo RL. Activation of TLR3 in keratinocytes increases expression of genes involved in formation of the epidermis, lipid accumulation, and epidermal organelles. The Journal of investigative dermatology. 2013;133(8):2031–40. Epub 2013/01/29. 10.1038/jid.2013.39 23353987 PMC3686920

[40] Belair C, Darfeuille F, Staedel C. Helicobacter pylori and gastric cancer: possible role of microRNAs in this intimate relationship. Clinical microbiology and infection: the official publication of the European Society of Clinical Microbiology and Infectious Diseases. 2009;15(9):806–12. Epub 2009/08/26. 10.1111/j.1469-0691.2009.02960.x . 19702585

[41] Hering NA, Luettig J, Krug SM, Wiegand S, Gross G, van Tol EA, et al Lactoferrin protects against intestinal inflammation and bacteria-induced barrier dysfunction in vitro. Annals of the New York Academy of Sciences. 2017;1405(1):177–88. Epub 2017/06/15. 10.1111/nyas.13405 . 28614589

[42] Lander HM, Grant AM, Albrecht T, Hill T, Peters CJ. Endothelial cell permeability and adherens junction disruption induced by junin virus infection. Am J Trop Med Hyg. 2014;90(6):993–1002. Epub 2014/04/09. 10.4269/ajtmh.13-0382 24710609 PMC4047760

[43] Hu S, Hosey KL, Derbigny WA. Analyses of the pathways involved in early- and late-phase induction of IFN-beta during C. muridarum infection of oviduct epithelial cells. PLoS One. 2015;10(3):e0119235 10.1371/journal.pone.0119235 25798928 PMC4370658

[44] MacIntyre A, Hammond CJ, Little CS, Appelt DM, Balin BJ. Chlamydia pneumoniae infection alters the junctional complex proteins of human brain microvascular endothelial cells. FEMS microbiology letters. 2002;217(2):167–72. Epub 2002/12/14. 10.1111/j.1574-6968.2002.tb11470.x . 12480099

[45] Wira CR, Fahey JV, Ghosh M, Patel MV, Hickey DK, Ochiel DO. Sex hormone regulation of innate immunity in the female reproductive tract: the role of epithelial cells in balancing reproductive potential with protection against sexually transmitted pathogens. Am J Reprod Immunol. 2010;63(6):544–65. Epub 2010/04/07. 10.1111/j.1600-0897.2010.00842.x 20367623 PMC3837356

[46] Prozialeck WC, Fay MJ, Lamar PC, Pearson CA, Sigar I, Ramsey KH. Chlamydia trachomatis disrupts N-cadherin-dependent cell-cell junctions and sequesters beta-catenin in human cervical epithelial cells. Infection and immunity. 2002;70(5):2605–13. Epub 2002/04/16. 10.1128/IAI.70.5.2605-2613.2002 11953402 PMC127927

[47] Xia M, Bumgarner RE, Lampe MF, Stamm WE. Chlamydia trachomatis infection alters host cell transcription in diverse cellular pathways. J Infect Dis. 2003;187(3):424–34. Epub 2003/01/29. 10.1086/367962 . 12552426

[48] Cotter TW, Ramsey KH, Miranpuri GS, Poulsen CE, Byrne GI. Dissemination of Chlamydia trachomatis chronic genital tract infection in gamma interferon gene knockout mice. Infection and immunity. 1997;65(6):2145–52. . 9169744 10.1128/iai.65.6.2145-2152.1997 PMC175296

[49] Darville T, Andrews CW Jr., Laffoon KK, Shymasani W, Kishen LR, Rank RG. Mouse strain-dependent variation in the course and outcome of chlamydial genital tract infection is associated with differences in host response. Infection and immunity. 1997;65(8):3065–73. . 9234755 10.1128/iai.65.8.3065-3073.1997 PMC175432

[50] Igietseme JU, Uriri IM, Kumar SN, Ananaba GA, Ojior OO, Momodu IA, et al Route of infection that induces a high intensity of gamma interferon-secreting T cells in the genital tract produces optimal protection against Chlamydia trachomatis infection in mice. Infection and immunity. 1998;66(9):4030–5. . 9712743 10.1128/iai.66.9.4030-4035.1998 PMC108481

[51] Ito JI, Lyons JM. Role of gamma interferon in controlling murine chlamydial genital tract infection. Infection and immunity. 1999;67(10):5518–21. . 10496942 10.1128/iai.67.10.5518-5521.1999 PMC96917

[52] Johansson M, Schon K, Ward M, Lycke N. Genital tract infection with Chlamydia trachomatis fails to induce protective immunity in gamma interferon receptor-deficient mice despite a strong local immunoglobulin A response. Infection and immunity. 1997;65(3):1032–44. . 9038313 10.1128/iai.65.3.1032-1044.1997 PMC175085

[53] Roan NR, Starnbach MN. Immune-mediated control of Chlamydia infection. Cell Microbiol. 2008;10(1):9–19. Epub 2007/11/06. 10.1111/j.1462-5822.2007.01069.x . 17979983

[54] Zhong G, Fan T, Liu L. Chlamydia inhibits interferon gamma-inducible major histocompatibility complex class II expression by degradation of upstream stimulatory factor 1. J Exp Med. 1999;189(12):1931–8. . 10377188 10.1084/jem.189.12.1931 PMC2192973

[55] Zhong G, Liu L, Fan T, Fan P, Ji H. Degradation of transcription factor RFX5 during the inhibition of both constitutive and interferon gamma-inducible major histocompatibility complex class I expression in chlamydia-infected cells. J Exp Med. 2000;191(9):1525–34. . 10790427 10.1084/jem.191.9.1525 PMC2213440

[56] Pirbhai M, Dong F, Zhong Y, Pan KZ, Zhong G. The secreted protease factor CPAF is responsible for degrading pro-apoptotic BH3-only proteins in Chlamydia trachomatis-infected cells. The Journal of biological chemistry. 2006;281(42):31495–501. Epub 2006/08/31. 10.1074/jbc.M602796200 . 16940052

[57] Heuer D, Rejman Lipinski A, Machuy N, Karlas A, Wehrens A, Siedler F, et al Chlamydia causes fragmentation of the Golgi compartment to ensure reproduction. Nature. 2009;457(7230):731–5. Epub 2008/12/09. 10.1038/nature07578 . 19060882

[58] Hickey DK, Patel MV, Fahey JV, Wira CR. Innate and adaptive immunity at mucosal surfaces of the female reproductive tract: stratification and integration of immune protection against the transmission of sexually transmitted infections. J Reprod Immunol. 2011;88(2):185–94. Epub 2011/03/01. 10.1016/j.jri.2011.01.005 21353708 PMC3094911

[59] Hu YJ, Wang YD, Tan FQ, Yang WX. Regulation of paracellular permeability: factors and mechanisms. Mol Biol Rep. 2013;40(11):6123–42. Epub 2013/09/26. 10.1007/s11033-013-2724-y . 24062072

[60] Minagar A, Long A, Ma T, Jackson TH, Kelley RE, Ostanin DV, et al Interferon (IFN)-beta 1a and IFN-beta 1b block IFN-gamma-induced disintegration of endothelial junction integrity and barrier. Endothelium. 2003;10(6):299–307. Epub 2004/01/27. . 14741845 10.1080/10623320390272299

[61] Kraus J, Ling AK, Hamm S, Voigt K, Oschmann P, Engelhardt B. Interferon-beta stabilizes barrier characteristics of brain endothelial cells in vitro. Annals of neurology. 2004;56(2):192–205. Epub 2004/08/05. 10.1002/ana.20161 . 15293271

