# Supplementary figures and images for: TLR3 deficiency exacerbates the loss of epithelial barrier function during genital tract Chlamydia muridarum infection
Source: PLoS One. 2019 Jan 9;14(1):e0207422. doi: 10.1371/journal.pone.0207422 (PMC6326510; doi:10.1371/journal.pone.0207422)

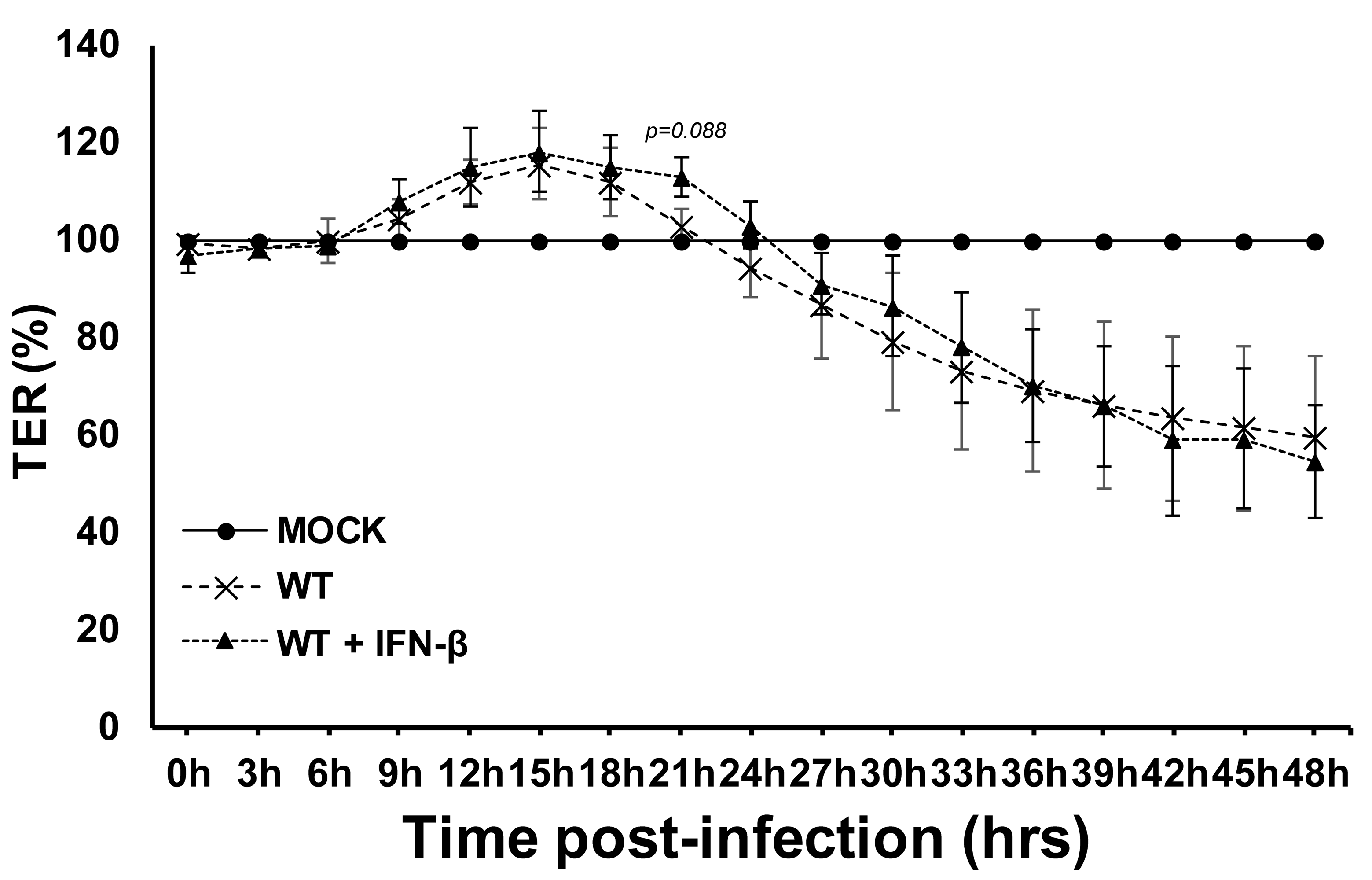

Supplement: S1 Fig — TER was measured every three hours in mock and C. muridarum infected: WT OE cells and WT OE cells pre-treated with 50U/ml IFN-β 1hr prior to infection. TER at each time-point is relative to Mock-infected controls of each respective OE cell condition set at 100%. Data are representative of 6 independent experiments. WT = wild-type OE cells. (TIF) [file pone.0207422.s001.tif]

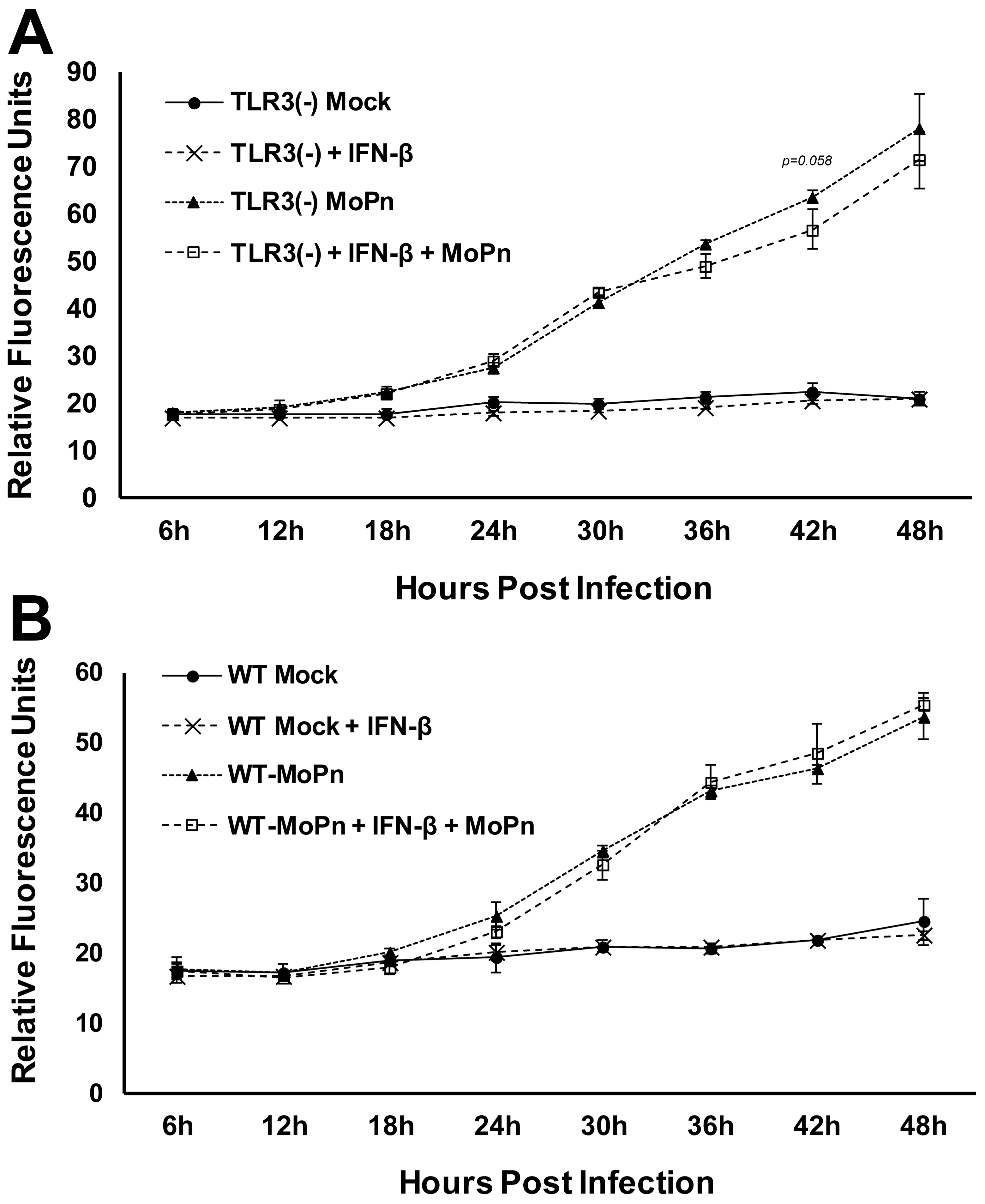

Supplement: S2 Fig — Macromolecular permeability assays were performed in (A) TLR3-deficient and (B) WT OE cell lines that were either untreated or pre-treated with 50U/ml recombinant IFN-β 1hr before being either mock-infected or C. muridarum-infected at an MOI of 1 IFU/ cell. Relative permeability was measured using a FITC-labeled dextran (70-kDa) probe. Samples were taken from the basolateral chamber of the transwell every 6hrs post-infection, and permeability was determined by increases in relative fluorescence compared to mock-infected controls. Data are representative of three independent experiments. WT = wild-type and TLR3(-) = TLR3-deficient OE cells. (TIF) [file pone.0207422.s002.tif]

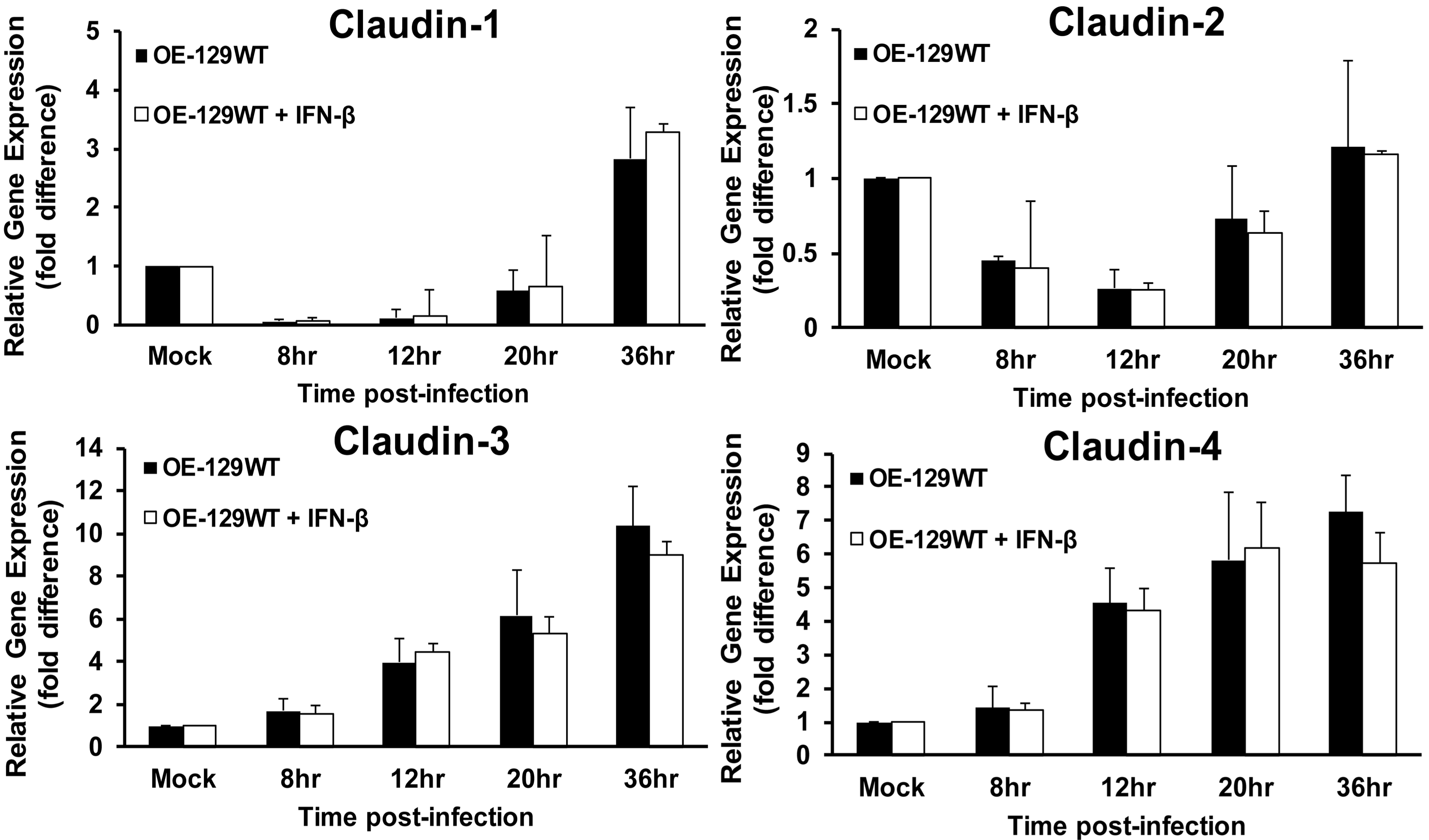

Supplement: S3 Fig — Gene expression levels of Claudins 1–4 were measured by qPCR at various times post-infection in C. muridarum infected WT OE cells that were either mock-treated or pre-treated with 50U/ml recombinant IFN-β 1hr before infection. Data are representative of three or more independent experiments. OE-129WT = wild-type OE cells. (TIF) [file pone.0207422.s003.tif]

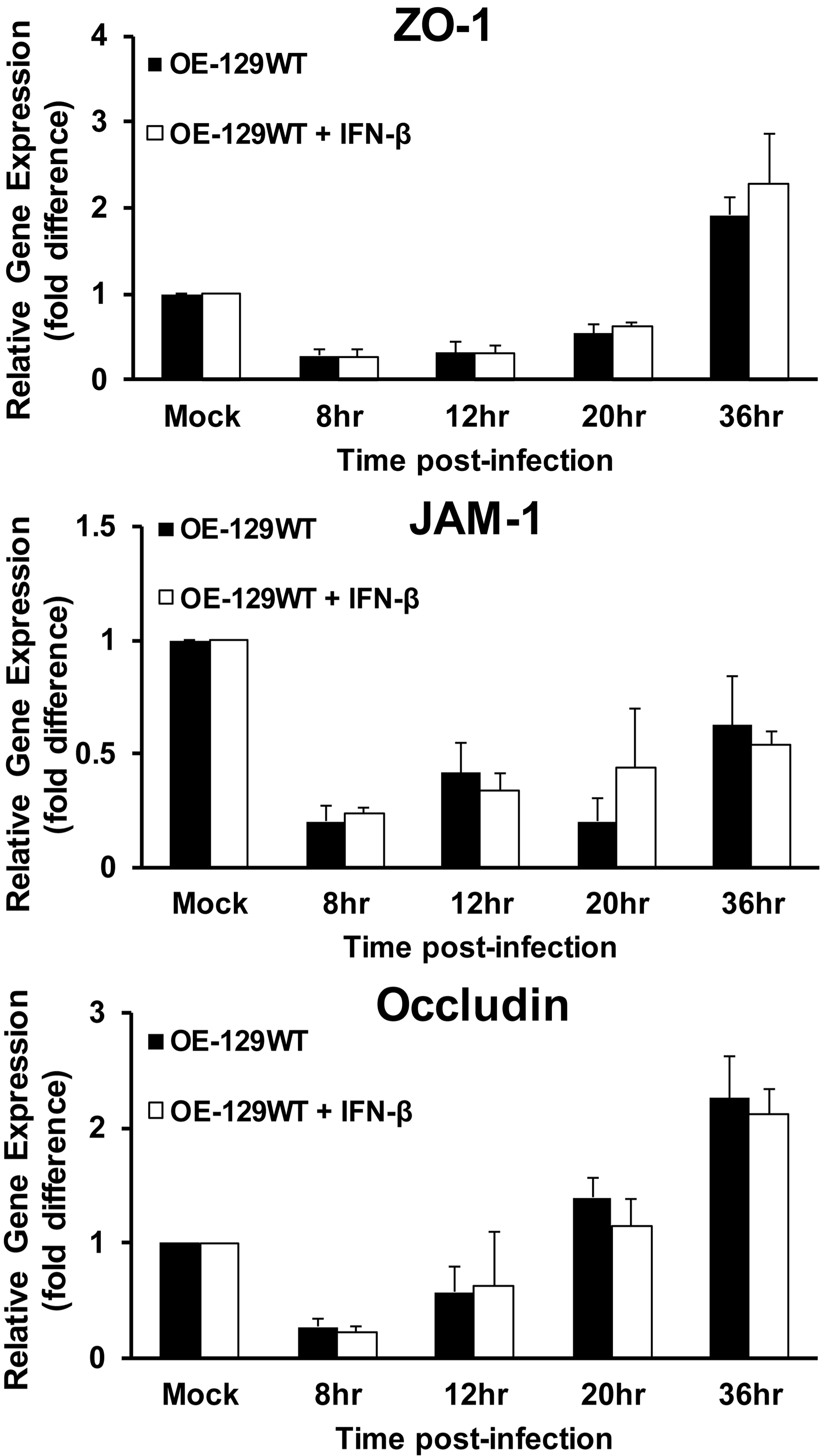

Supplement: S4 Fig — Gene expression levels of ZO-1, JAM-1, and occludin were measured by qPCR at various times post-infection in C. muridarum infected WT OE cells that were either mock-treated or pre-treated with 50U/ml recombinant IFN-β 1hr before infection. Data are representative of three or more independent experiments. OE-129WT = wild-type OE cells. (TIF) [file pone.0207422.s004.tif]
